# Verbal Episodic Processing in Newborns

**DOI:** 10.7554/eLife.109096

**Published:** 2026-06-24

**Authors:** Emma Visibelli, Ana Fló, Eugenio Baraldi, Silvia Benavides-Varela

**Affiliations:** 1 Department of Developmental Psychology and Socialisation; Padova Neuroscience Center, University of Padua Padova Italy; 2 Neonatal Intensive Care Unit, Department of Women’ and Children's Health, University Hospital of Padova Padova Italy; New York University Shanghai China; Peking University China

**Keywords:** verbal memory, speaker identity, functional near- infrared spectroscopy, acoustic variability, newborns, Human

## Abstract

During the first period of life, human infants rapidly and effortlessly acquire the languages they are exposed to. Although memory is central to this process, the nature of early verbal memory systems, and the factors that determine retention and forgetting, remain largely unknown. Behavioral and brain measures have demonstrated memory formation in newborns. However, word traces fade in the face of acoustic overlap, leading to interference and forgetting. Here, we investigate whether speakers' identity changes facilitate the separation into distinct acoustic episodes and the creation of non-overlapping verbal memories. Newborns (0–4 days-old) were tested in a familiarization-interference-test protocol, while neural cortical activity was recorded using functional Near-Infrared Spectroscopy (fNIRS). The results showed higher neural activation to novel words than to familiar ones during the test phase, indicating that the infants recognized the familiar words despite potentially interfering sounds. The recognition response was measured over the left inferior frontal gyrus (IFG) and superior temporal gyrus (STG) areas known to be crucial for encoding auditory information and language processing. The neural response also included the right IFG and STG, involved in interpreting vocal social cues and speaker recognition. The results indicate that speaker identity is a key feature in the formation of verbal memories from birth, facilitating separability, possibly through early source–content binding (i.e. what–who), a precursor to fully mature episodic memory.

## eLife digest

When we remember events, we often do not just recall what happened, but also where and when it was, and who was there. This is because human memory tends to merge different features of experiences into whole episodes rather than store them as separate items. Combining all these features is what makes memories stronger and easier to remember in the long term, shaping who we are and how we act.

Our earliest episodic memories probably go back to the first years of life. But linguistic memory may develop earlier. Infants start learning a language before they can speak. By four months, they can respond to their own name and by six months, they can recognise common words. This suggests that they already possess the ability to store and retrieve linguistic information. But what is the nature of these earliest memories, and when are they first formed?

To find out more, Visibelli et al. tested brain signatures of episodic-like, linguistic memories in infants a few hours after birth. They designed a task to examine verbal memory formation in the presence of different speakers. Newborns, while lying in their hospital cribs, were exposed to a word pronounced by one speaker and then listened to the same or a different word three minutes later, after having been exposed to interfering words pronounced by another speaker.

Visibelli et al. investigated whether a change in who produces the words (the *who*) helps newborns create separate, retrievable memory traces for the words (the *what*). The researchers used a non-invasive method known as functional Near-Infrared Spectroscopy, which uses near-infrared light to monitor changes in brain oxygenation.

The recordings showed that newborns’ brains responded differently to a word they had heard before than to one they had not. This difference in response indicates that the brain distinguishes the familiar word from a new one - a sign that a memory had been stored and retrieved. Crucially, these memories were maintained only when different speakers produced both the familiar and interfering words. When recognition was successful, brain regions involved in speech and voice processing became active, suggesting that infants were not only processing the words themselves, but also who was speaking. This suggests that, at birth, newborns readily link words to speaker identity, encoding both *what* is being said and *who* is speaking.

The findings of Visibelli et al. open new directions for understanding memory and language development from the very first hours of life. The data suggest that speaker identity is a key feature of speech, enabling episodic-like memories of word sounds from birth and offering evolutionary advantages at the outset of human communication. They also raise the possibility that difficulties with feature binding (when the brain combines different attributes of an object or sound) may be detectable at a very early stage – before any language difficulties become apparent – thereby creating new opportunities for early identification and intervention.

## Introduction

Word recognition entails processing and integrating various linguistic features, such as phonological content, along with contextual or indexical information, like speaker identity, accent, and emotional content, which are crucial for communication. Theoretical approaches to speech representation hold contrasting views on the role of indexical features in word recognition. Abstractionist models assumed that variability needs to be normalized or stripped away so that speech sounds could be recognized (e.g. Halle, 1985; McClelland and Elman, 1986; Norris et al., 2000; Pisoni and Luce, 1987). Episodic or exemplar approaches adopt an alternative perspective, assuming that memories of linguistic utterances are bound to indexical information (e.g. Goldinger, 1996; Nygaard et al., 1994; Palmeri et al., 1993). The balance between forming exemplar memories and creating normalized word prototypes is crucial during language acquisition. Indexical information may aid in distinguishing memories, while abstract representations are necessary for generalization. However, how infants encode language as they develop is still not well understood.

When encoding word forms, young infants remember not just the words themselves but also specific indexical properties such as the speaker (Houston and Jusczyk, 2000), stress, amplitude, and affect (Singh et al., 2004; see Heugten et al., 2015). However, their learning is context-dependent: low-variability conditions promote the learning of specific examples (Houston and Jusczyk, 2000; Jusczyk and Aslin, 1995; Singh et al., 2004) and high-variability conditions facilitate the learning of abstract word prototypes (Singh, 2008). Current models of infant language comprehension (Jusczyk, 1997; Werker and Curtin, 2005) propose that in early stages, infants match specific sounds to stored instances of words and subsequently generate abstract word prototypes. In line with this, we hypothesize that speaker changes play a critical role in verbal memories' formation at birth by providing indexical information for memory separation.

Verbal memory formation at birth is not well understood. Vast research on language processing supports the storage of both linguistic and speaker-specific information in newborns. Neonates readily distinguish phonetic changes (Cheour-Luhtanen et al., 1995; Dehaene-Lambertz and Pena, 2001), extract words from continuous speech (Fló et al., 2022; Fló et al., 2019), and detect speech structure (Benavides-Varela and Gervain, 2017; Gervain et al., 2008; Martinez-Alvarez et al., 2023), even amidst variability in speakers (Fló et al., 2025; Mahmoudzadeh et al., 2013). Newborns also react to indexical features such as between-accent differences (Giordano et al., 2021) and are particularly sensitive to familiar voices (DeCasper and Fifer, 1980; Mehler et al., 1978; Spence and Freeman, 1996). Moreover, phonological processing is lateralized to the left hemisphere, while voice-related information shows right lateralization already in young infants (Blasi et al., 2011; Spence and Freeman, 1996; see review Grossmann et al., 2010). While these findings support normalized phonological representations and parallel processing of phonological and contextual features, it remains unclear how these features are integrated to form verbal memories at birth and how they can determine memory formation or forgetting.

Benavides-Varela and colleagues used functional near-infrared spectroscopy (fNIRS) to investigate the formation of word memories at birth, including the brain areas supporting this cognitive capacity and the factors that determine their loss or retention. The authors found that newborns familiarized with a two-syllable word sound (hereafter referred to as word) show a recognition response after a few-minute-long retention period, which was characterized by decreased activity toward the familiar word and increased response to a novel word over temporal, frontal, and parietal areas (Benavides-Varela, 2012; Benavides-Varela et al., 2011a; Benavides-Varela et al., 2012). This research also indicated that under some circumstances, newborns’ memories appear fragile and highly vulnerable to interference. For example, recognition does not persist when neonates hear another word produced by the same speaker during the retention period. Interestingly, unlike speech, instrumental music presented during this retention phase does not interfere with the familiar memory trace (Benavides-Varela et al., 2011a). The phenomenon could be partly explained by retroactive interference, which occurs when novel information disrupts the retention of previously learned items (Müller G. and Pilzecker, 1900). One factor that may influence retroactive interference is the degree of neural overlap between the information to be encoded and the interfering stimuli. Since instrumental music and speech processing recruit partially distinct brain areas (in adults: Peretz et al., 2015; Zatorre et al., 2002; infants: Dehaene-Lambertz et al., 2002; newborns: Kotilahti et al., 2010; Perani et al., 2010), this could explain the absence of music-speech interference. However, if this were the sole factor determining interference, speech-speech retroactive interference would render language learning impossible in real-life conditions. Here, we propose a complementary explanation for the retroactive interference described in previous studies: various features may be integrated to assess the similarities or differences between two auditory events, facilitating the separability of newly arriving information and, therefore, memory storage. Specifically, non-phonological information in speech, such as a speaker change, could serve as indexical information—acting as markers that signify the end of one event and the beginning of another—thereby facilitating the contrast and separability of verbal memories early in life. According to this hypothesis, the presence of speech during the retention period will not always lead to forgetting.

To test our hypothesis, we implemented a protocol derived from the work of Benavides-Varela et al., 2012; Benavides-Varela et al., 2011a. Newborns were first familiarized with a pseudoword produced by a single speaker. Immediately after, they were exposed to an interfering word. Then, in the test, the familiarization word or a completely novel word was presented. Like in Benavides-Varela et al., the interfering, the familiar, and the novel words had similar intensity, duration, pitch, syllable structure, etc. (see Appendix 1—table 1). Instead, the familiarization was reduced from ten to five blocks, and the retention interval increased from two to three minutes, making the paradigm more challenging. These methodological adjustments allow for a meaningful comparison with previous studies: if newborns forgot the familiarization word when an interfering word was presented as in Benavides-Varela et al., 2011a, they are expected to forget it also under our more challenging paradigm. However, unlike the previous work, the interfering word here was uttered by a different speaker. We hypothesize that if the voice distinction promotes memory separation, there should be a differential hemodynamic response between the familiar and a novel word in the test phase, signaling recognition. Instead, a failure in word recognition would reveal that a voice change is not sufficient to overcome the interference effect previously reported with this paradigm. We included 32 neonates in the final analysis, a number comparable to the 28 infants tested in the previous experiment showing interference (Benavides-Varela et al., 2011a), which should warrant enough statistical power. A remarkable difference between this and previous studies is the use of a within-subject design with two familiarization-interference-test sequences (one testing responses to novel words and the other to familiar words). This design controls for differences in anatomy, physiology, and brain activity across individuals while increasing statistical power.

## Results

**Figure 1.**
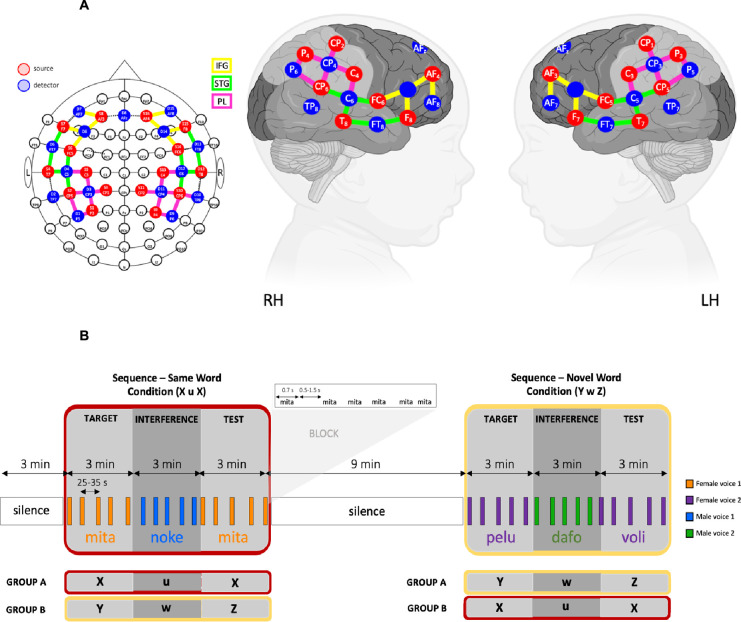
Experimental protocol. (**A**) Illustrative 42-channel fNIRS Montage. S (red)=source, D (blue)=detector. Placement indicated using the 10–10 standard EEG system. Regions of interest are indicated in yellow = inferior frontal gyrus (IFG), in green = superior temporal gyrus (STG), and in pink = parietal lobes (PL). (**B**) Familiarization-interference-test paradigm. Each subject was tested in two sequences separated by 9 min of silence: in one sequence, newborns heard the same word during familiarization and test (same-word condition; X u X), and in the other sequence, a novel word was presented during the test phase (novel-word condition; Y w Z). The order of the conditions, the words, and the voices used in the different phases were counterbalanced across participants.

In this paradigm, responses are expected to change over time due to habituation and recognition dynamics. Accordingly, it is not appropriate to average responses across blocks belonging to the familiarization and test phases. Block-level analyses were thus conducted using Linear Mixed Models (LMM), which are well-suited to handle missing values. This approach was necessary because each subject provides a unique instance for each block, which inevitably leads to missing values in the dataset—for example, when a motion artifact renders an entire block invalid for that subject. We used the hemodynamic response over each block and the six Regions of Interest (ROIs) covered by the probe as the dependent variable. We decided to analyze the data at the ROI level, since channel-level analysis is potentially more susceptible to optodes placement differences. Moreover, channel-level analysis increases the number of comparisons in a protocol that already needs to compare activation over multiple blocks. Nevertheless, analyzing the data at the channel level yielded similar results (see Appendix 1—table 2). The ROIs were symmetric between hemispheres and included the inferior frontal gyrus left and right (IFGl, IFGr), the superior temporal gyrus left and right (STGl, STGr), and the parietal lobe left and right (PLl, PLr; Figure 1A). We modeled fixed effects (e.g. condition: *same* or *novel*) nested within the block number and the ROIs, while including participants as random effects. Each such model indicates whether there are significant fixed effects within each block and ROI, without the need to correct for multiple comparisons for the number of blocks and ROIs. Only results for oxy-hemoglobin (HbO) are presented here. Results for deoxy-hemoglobin (HbR) were less clear and are presented in the SI (Appendix 1—figure 2).

### Activity during familiarization

**Figure 2.**
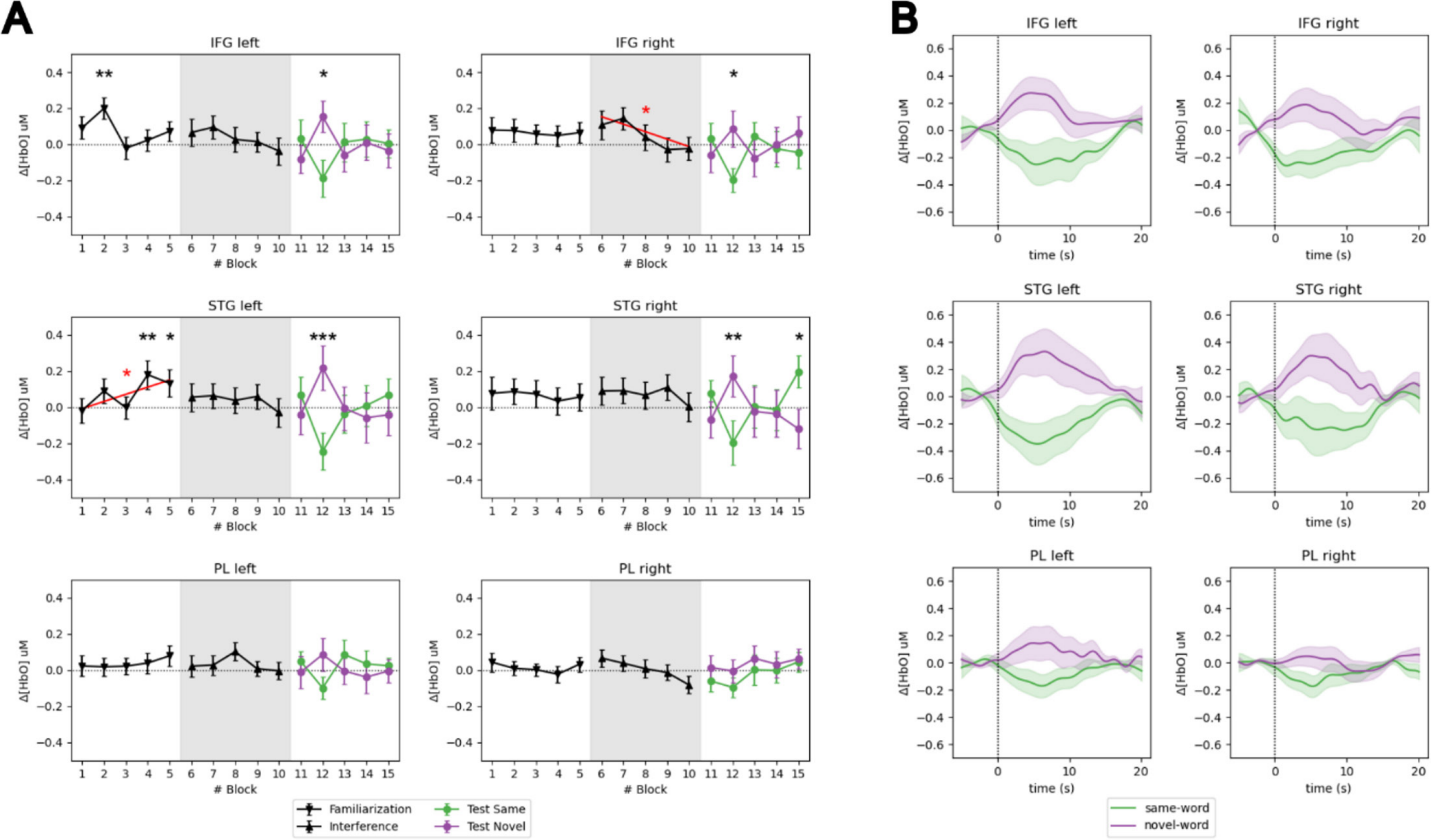
Standard recognition response with decreased activity for the familiar words and increased activity for the novel words in the test phase. (**A**) Mean activity for HbO per block during the familiarization, interference, and test phases. Error bars represent the standard errors. The black continuous line depicts responses averaged across all participants and conditions. The same-word condition (green) and the novel-word condition (purple) are plotted in the test phase. The black asterisks during the familiarization and interference phases indicate that the response differed from zero. The red lines indicate a significant linear trend, as indicated by the red asterisks. Black asterisks during the test phase indicate significant differences between conditions. (**B**) HRFs for HbO during the second block of the test phase, when relevant differences were observed between conditions. Shaded areas represent the standard error.

To assess potential habituation and novelty effects commonly observed in fNIRS data, we first tested whether the activity differed from zero by fitting the LMM *act ~–1+block:ROI+(1|sub*) during the familiarization blocks. This model provides one coefficient for each ROI and block (*β(ROI_j_, block_i_*)) representing the activation. The model showed a positive activation in block 2 within left IFG (*β(IFGl, b2)=0.194, SE = 0.064, p=0.024*) and during blocks 4 and 5 within left STG (*β(STGl, b4)=0.173, SE = 0.065, p=0.008; β(STGl, b5)=0.128, SE = 0.063, p=0.044*) (Figure 2A).

Additionally, we tested for linear changes in activity by fitting the LMM: *act ~–1+ROI + ROI:blocknumber+(1|sub)*, with *blocknumber* coded from 0 to 4. This coding scheme allows the intercept term for each ROI to represent activity in the first block, while the corresponding slope term captures any linear change in activity across blocks.

The model showed a significant intercept in the left IFG (intercept = 0.1105, SE = 0.0535, p=0.040), indicating an initial positive activation and a significant positive slope in the left STG (slope = 0.0396, SE = 0.018, p=0.029), denoting a sustained increase in activity in this area (Figure 2A). An analogue analysis for the interference phase is presented in the SI (Appendix 1—figure 4).

### Word recognition

We assessed recognition responses in the test phase by testing whether the activation pattern differed between the familiar and novel words. We employed an LMM, including condition as a fixed factor nested within the ROIs and blocks of the test phase *act ~–1+block:ROI +block:ROI:condition+(1|sub)*. Such a model provides, for each ROI and block, one coefficient quantifying activation in one condition and another quantifying the difference between conditions – thus, crucial for evaluating word recognition. The model showed a significantly higher activation during the second block of the test phase for the *novel-word* than the *same-word* condition over IFG and STG (*β(IFGl, b2)=0.322, SE = 0.133, p=0.015; β(IFGr, b2)=0.265, SE = 0.133, p=0.045; β(STGl, b2)=0.443, SE = 0.133, p=0.0009; β(STGr, b2)=0.348, SE = 0.133, p=0.009*). Instead, activity was higher for the *same-word* than the *novel-word* condition in the fifth block over STG right (*β(STGr, b5)=−0.320, SE = 0.127, p=0.012*). To investigate the presence of hemispheric differences in the main effect of condition revealed by the primary analysis, we ran an LMM restricted to the second block and the IFG and the STG separately (act ~cond*hemisphere+ (1 | sub)). We found no significant effects of hemisphere or interaction, neither over IFG nor on STG (p>0.1; see Figure 2A–B).

### Effects of the sequences order

In our within-subject design, group A first completed the same-word condition (X u X) and later the novel-word condition (Y w Z), while group B did the opposite (Figure 1B). Thus, the first sequence might influence the processing of the second sequence, potentially leading to differences between sequences and groups.

We looked for differences during the familiarization and interference phases by fitting an LMM contrasting (1) first and second sequence, (2) groups within the first sequence, and (3) groups within the second sequence. The contrasts were nested within blocks and ROIs, such that, for each ROI and block, a coefficient was fitted for each contrast (see details in SI). The model showed higher activation during the second than the first familiarization in the first block over IFG and STG (*β(IFGl, b1, contrast 1)=−0.254, SE = 0.191, p=0.033; β(STGl, b1, contrast 1)=−0.249, SE = 0.191, p=0.036; β(STGr, b1, contrast 1)=−0.322, SE = 0.191, p=0.0069*; Appendix 1—figure 3). Differences between groups were weak and restricted to higher activation in group B than A in the first block of the first sequence over the left STG (*β(STGl, b1, contrast 2)=−0.368, SE = 0.182, p=0.043*) and on the first block of the second sequence over the right STG *(β(STGr, b2, contrast 3)=−0.355, SE = 0.175, p=0.042*). Considering the small number of data points per group and sequence, these differences are likely due to noise. See in SI the analysis for the interference phase (Appendix 1—figure 4).

Given the differences in activation between the first and second familiarization phases, we quantify linear changes in activity as we did previously, but separately for each familiarization sequence. For the first familiarization, the model showed a significant increase in activity in the left and right STG and left PL (p<0.05), while during the second familiarization, the activity was higher than zero in the first block and decreased with block number on the right STG and IFG (p<0.05; detailed results are presented in Appendix 1—figure 3).

To check for differences between the two groups during the testing phase, we fitted an LMM contrasting (1) the *same-word* and *novel-word* conditions, (2) the groups within the *same-word* condition (i.e. *same-word* presented in group A, thus, sequence 1, or group B, thus, sequence 2), and (3) the groups within the *novel-word* condition (i.e. *novel-word* presented in group A, sequence 2, or group B, thus, sequence 1). The contrasts were nested within blocks and ROIs, yielding a coefficient for each contrast within each ROI and block. In agreement with the overall results obtained when merging the two groups, the model showed a significant main effect of condition during the second block over IFG and STG (*β(IFGl, b2, contrast 1)=0.334, SE = 0.131, p=0.011; β(IFGr, b2, contrast 1)=0.293, SE = 0.131, p=0.026; β(STGl, b2, contrast 1)=0.459, SE = 0.131, p=0.00050; β(STGr, b2, contrast 1)=0.367, SE = 0.131, p=0.0053*), and during the fifth block over right STG (*β(STGr, b5, contrast 1)=−0.358, SE = 0.128, p=0.0051*). No significant differences were observed between groups (sequences) for the *same-word* condition (p>0.05). However, the model showed significant group differences for the *novel-word*. Activation was higher for the *novel-word* in group A (*novel-word* in sequence 2) than in group B (*novel-word* in sequence 1) in the second block over right IFG (*β(IFGr, b2, contrast 3)=0.538, SE = 0.183, p=0.0031*) and in the third block over left and right STG (*β(STGl, b3, contrast 3)=0.394, SE = 0.182, p=0.031; STGr: β=0.533, SE = 0.182, p=0.0036*). Instead, activity for the *novel-word* was higher for group B than A in the fourth block over IFG and STG (*β(IFGl, b4, contrast 3)=−0.443, SE = 0.198, p=0.025; β(IFGr, b4, contrast 3)=−0.390, SE = 0.198, p=0.049; β(STGr, b4, contrast 3)=−0.403, SE = 0.198, p=0.042*).

**Figure 3.**
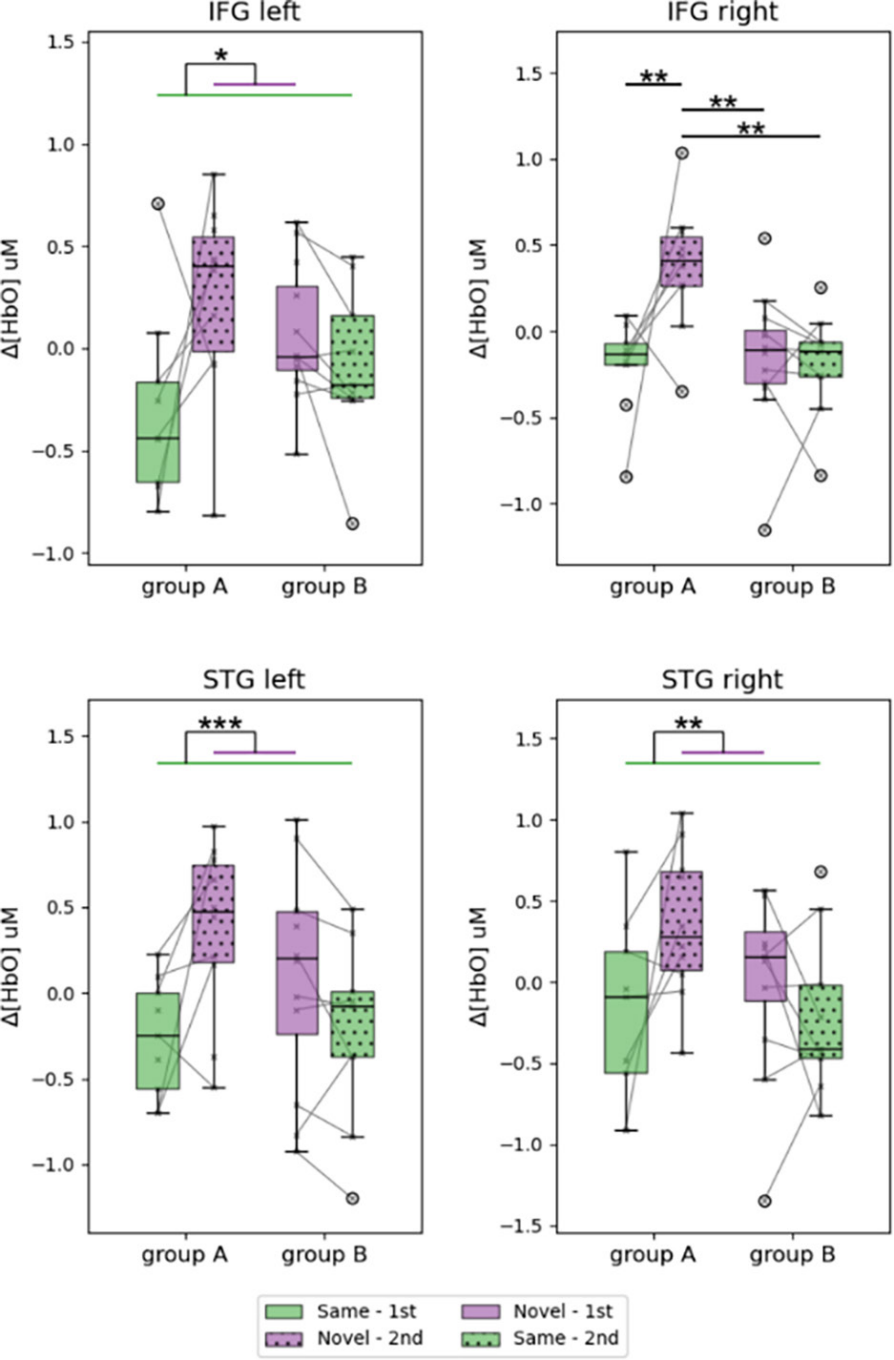
Differences in the response across groups during the second test block. Boxplots represent the mean HbO activity during the second block of the test phase, separated by condition (same = green, novel = purple), group (A or B), and sequence (first = full pattern or second = dotted pattern). Whiskers of the boxplot are defined based on 1.5 times the interquartile range, and data points outside these limits are plotted as circles. Asterisks indicated significant differences between conditions or groups. A significant effect of conditions was observed in the left IFG and the left and right STG, reflecting recognition in both groups. Instead, an interaction effect was present in the right IFG with higher activity in the *novel-word* condition for group A.

This analysis confirms that both groups show a consistent recognition response (higher activity for the *novel-word* than the *same-word*) in the second block, over left and right STG, and left IFG. In addition, it indicates a more complex pattern of activation in the right IFG, with an interaction between condition and group. To better understand the effect, we performed Tukey’s multiple-comparison test. Results showed higher activation for *novel-word* in group A than all the other conditions (*novel-word* in group B: p=0.007, *same-word* in group A: p=0.0096, and *same-word* in group B: p=0.0073), confirming that the difference between conditions on the right IFG during the second block was driven by group A (Figure 3).

## Discussion

### The role of variability in early memory processes

In the current study, we investigated the conditions that promote the formation of separate memory traces of linguistic stimuli at birth. We observed a persistent neural signature of recognition, namely a differential response between the familiar and novel words, by introducing a change in the speaker uttering the interference word. In Benavides-Varela et al., 2011a, word recognition in neonates vanished when they heard an interference word pronounced by the same speaker who uttered the familiarization word. We hypothesize that the shared voice feature could have increased perceived acoustic overlap (Apfelbaum and McMurray, 2011), thereby causing interference. In our study, the presence of a new speaker might rather act as a conspicuous cue signaling the beginning of a new acoustic episode and facilitating the separation of linguistic memory traces.

These results demonstrate that, under certain conditions, newborns can retain verbal memories even when the language networks continuously receive new verbal information, as in real life. These results are in line with episodic models of early speech perception, assuming that infants initially store words in an instance-specific fashion comprising both phonological details and speaker identity (Jusczyk, 1997; Werker and Curtin, 2005). Furthermore, the findings extend these models by offering empirical evidence of episodic encoding in newborns: early word-form representations are, at least to some extent, linked to the acoustic realization of the word and, when it comes to early signal-to-word form mappings, the newborn brain attributes significant relevance to voices. The speaker’s identity may thus represent a critical distinguishing factor essential for early communication and memory. Forgács et al., 2022 recently showed that alternation between female and male voices, combined with partial variability in the syllable stream, elicited greater activation in the left fronto-temporal regions. This finding suggests that the facilitation of verbal human memory in newborns might also be related to the heightened neural activation associated with communicative attribution. In this view, infants may interpret such vocal alternations as indicative of a communicative exchange, thereby enhancing their ability to segregate and store the pseudo-words presented as stimuli.

These findings speak to the relevance of certain cues in the sequential processing of speech input, but do not inform us about the possibility that newborns can handle indexical variation (i.e. speaker changes or changes in intonation and emotional content) during the presentation of the word in the familiarization phase or recognize the familiar word irrespective of possible indexical variations in the test. There are some hints in the literature suggesting that this might be the case. Newborns robustly encode words presented in concomitance with other words, suggesting that word-memories can be formed in the face of input variability (Benavides-Varela et al., 2012). Moreover, newborns show recognition of pseudowords despite prosodic differences (Fló et al., 2019) and compute regularities over phonetic content, disregarding the voice content (Fló et al., 2025). Thus, it is possible that if a variety of diverse tokens are presented during learning, a robust and generalizable representation could emerge as early as birth. While this question lies beyond the scope of the present study, it could provide additional insights into early word recognition processes.

### Signature of word recognition and areas recruited for memory retrieval

In the current study, we observed the typical recognition response characterized by an increase in activity for the novel word and a decrease for the familiar one, consistent with previous studies using a similar paradigm. Although the fNIRS system and optodes positioning slightly differed from those of previous studies (e.g. Benavides-Varela and colleagues’ system covered more prefrontal areas, whilst our configuration only reached the IFG), the activation pattern in the temporal and frontal areas is generally consistent across studies. In the present study, the effect was bilateral over the IFG and STG, known to play a crucial role in language processing and in interpreting vocal social cues in the left and right hemisphere, respectively. In particular, left frontal regions, including the IFG, are associated with processing, retrieving, and manipulating phonological information (e.g. Bunge et al., 2001; Hickok and Poeppel, 2007; Novick et al., 2010; Thompson-Schill et al., 1997), and the left STG plays a crucial role in phonological and semantic processing (Hickok and Poeppel, 2007) by encoding fast temporal (phonetic) information (DeWitt and Rauschecker, 2012; Mesgarani et al., 2014; Zatorre and Belin, 2001) and integrating auditory information within verbal memory (Cabeza and Nyberg, 2000). Conversely, speaker recognition relies primarily on a right-lateralized network (Mathias and Kriegstein, 2014), with the right IFG and STG essential for processing prosody, rhythm, and vocal social cues such as emotional state and intent (Agus et al., 2017; Belin et al., 2000; Belin et al., 2002; Bodin et al., 2018; Fecteau et al., 2004; Pernet et al., 2015; Wildgruber et al., 2006; Zatorre et al., 2002).

Precursors of the same organization and hemispheric specialization seem to be in place early on in life (Dehaene-Lambertz and Baillet, 1998; Telkemeyer et al., 2009), including the activation of left fronto-temporal areas associated with language processing (Alexopoulos et al., 2021; Alexopoulos et al., 2022; Dehaene-Lambertz et al., 2002; Peña et al., 2003) and functional specialization of the right STS for voice processing (Blasi et al., 2011; Cheng et al., 2012; Grossmann et al., 2010; Schönwiesner et al., 2005; Simon et al., 2009). The different responses we observed between new and familiar words after a three-minute retention period align with the retrieval of the verbal memory. Therefore, the bilateral concurrent responses over the IFG and STG suggest that linguistic and non-linguistic features of the word contribute to the recognition response in this context.

### Timing of the response: word recognition in the second block of the test phase

Factors including experimental design and stimulus complexity are known to influence hemodynamic responses in newborns and infants across tasks (Issard and Gervain, 2018). In this paradigm, an interplay between familiarization length and the presence of interfering sounds might determine when the differential response between a novel and a familiar stimulus emerges. In simple experiments, recognition is detected in the first block of the test when a single identical word is repeated over 6 min in the familiarization and when no interfering sounds are presented during the retention interval (e.g. Benavides-Varela et al., 2011a). In more complex designs, the recognition was delayed to the second block when an interfering word alternates with the to-be-remembered word during encoding (Benavides-Varela et al., 2012). Similarly, in the current study, the recognition response emerged in the second block of the test phase when an interfering word sound was presented during the retention phase. Thus, while newborns can recognize word sounds under complex conditions, facing these challenges influences the timing of the recognition response in the test phase, requiring additional cues or extended processing time for activation.

### Familiarization phase

Stable activity was registered with no obvious attenuation of the neural response over the 3 min in the familiarization phase. This general pattern was observed in most areas, but in the left STG, where the neural response showed repetition enhancement over time. Neural suppression (habituation) or enhancement, while expected in the context of repeated stimuli, is not consistently found across fNIRS studies in infants and newborns. Various factors may influence hemodynamic patterns over time. First, some studies using a protocol similar to ours found habituation over the left frontal areas in newborns when target words are presented in ‘ecological conditions’, that is, interleaved with other words (Benavides-Varela et al., 2017). By contrast, habituation is not reported when the familiarization is homogeneous (Benavides-Varela et al., 2011a), as in the current study. This suggests that the amount of information present during the learning phase modulates newborns’ fNIRS neural dynamics. The role of stimulus complexity has also been demonstrated in fNIRS studies of rule-learning in newborns. While highly variable speech sequences elicited left-lateralized repetition enhancement across blocks for ABB artificial grammar and no variations for ABC grammar (Gervain et al., 2008), simpler stimuli and presentation conditions (blocked rather than interleaved) evoked a stable response for the simpler ABB grammars and a repetition enhancement effect over time for ABC grammars (Bouchon et al., 2015). Second, methodological factors, such as the frequency and number of stimulus repetitions, are known to influence the habituation (Rankin et al., 2009). Thus, the sparse stimuli presentation typical of fNIRS block-designs (with stimuli followed by periods of 20–25 s of silence), along with the reduced number of blocks employed in the present study, may have also contributed to the patterns observed. Third, (Katus et al., 2023) recently tested habituation to a female voice in 1-month-old (asleep), 5-month-old (awake), and 18-month-old (awake) infants. They found that habituation began to emerge at 5 months and became strong by 18 months. Similarly, another study revealed stronger effects of habituation in 8-month-old awake infants compared to 5-month-olds (Lloyd-Fox et al., 2019). Altogether, these studies show that developmental changes and sleep state influence habituation as measured by fNIRS. It is therefore likely that all these factors (i.e. stimulus variability, stimulus frequency, duration of familiarization, participants’ age, and behavioral state) modulated the responses observed in the current study. Future research should carefully control these variables to further explore their role in learning and memory formation at birth.

### Cross-phase associations

A question raised by the present findings concerns how neural activity during the familiarization, interference, and test phases may relate to one another. The current study was not designed to test specific hypotheses about cross-phase dependencies, and therefore any such integration must remain descriptive. Nevertheless, the patterns of activity varied across ROIs, suggesting that encoding- and retrieval-related processes may interact in a region-specific manner. One illustrative example is the left STG, which showed a marked increase in activity during familiarization and a differential response between familiar and novel words in the earlier test blocks, which is consistent with a relationship between responses across phases within this ROI. By contrast, other regions, while also exhibiting a differential response at test, did not show comparably pronounced or systematic changes during the familiarization phase. In this context, converging evidence from developmental fNIRS work illustrates how such cross-phase dependencies can be revealed using connectivity-based approaches. For example, Benavides-Varela et al., 2017 observed a habituation-like hemodynamic response during encoding in left-frontal regions accompanied by progressive interactions between temporal and left-frontal regions. These interactions then served the recognition response in right-frontal and right-parietal regions, with connectivity from temporal areas emerging selectively for familiar items. The present data, based on univariate activation patterns, do not allow us to establish a direct causal link between activity during familiarization and subsequent test responses. Future work could further characterize how information is distributed across regions during familiarization, interference, and test, and how these patterns contribute complementary information to subsequent retrieval. Such approaches may also help clarify whether and how activity in specific regions during encoding predicts later recognition responses.

A further issue that merits discussion concerns the absence of an increase in activity during the interference phase. At first glance, this might seem at odds with the presence of a robust differential response in the test phase. However, the test phase engages memory recognition processes that rely on comparison of the incoming stimuli with stored representations and may therefore elicit a more sustained and detectable hemodynamic response than a mere acoustic change. Accordingly, a plausible explanation for this pattern relates to the temporal dynamics of the hemodynamic response, which in newborns may be less sensitive to transient sensory novelty than to the functional demands of higher-level processing. This interpretation is consistent with developmental evidence showing clearer neural responses to novel speech streams at later ages than in younger infants (e.g. at 3 months in Nakano et al., 2009; at 5 and 18 months but not at 1 month in Katus et al., 2023), suggesting that the detectability of sensory novelty effects by means of fNIRS possibly increases with maturation.

### Habituation, recognition, and novelty detection differences between groups

When interpreting the patterns in the familiarization, it is important to consider baseline activity. This consideration is especially relevant in within-subject designs, as the responses in the second session might be influenced by what newborns experienced in the first session. Our analysis captured these effects by showing higher activity in the first block of the second familiarization sequence than in the first. These results likely reflect a novelty response since all participants in the second familiarization session heard a new speaker pronouncing a completely novel word. At the same time, it provides evidence that newborns can retain information from the first session over a 9-min silent pause, allowing them to compare previously experienced episodes with newly encountered ones. These baseline differences result in distinct patterns over time: initial stronger activity followed by attenuation over blocks in the second sequences, while significant enhancement of the hemodynamic response is observed in the first sequences (Appendix 1—figure 3A).

The within-subjects design also offers a valuable opportunity to investigate the responses to familiar and novel words when infants first heard a familiar word at the test, followed by a novel one in the second sequence, or vice versa. Notably, the main novel/recognition response over left and right STG and left IFG was consistent across groups. In addition, a group modulation was observed over the right IFG: Group A (which encountered the novel word condition during the second testing sequence) showed a stronger response in the right IFG than Group B (which experienced it during the first testing sequence). This effect, although unexpected, could be explained by the number of phonological or speaker changes newborns experienced until the novel stimulus was presented. Indeed, while the novel word corresponds to the fourth change for newborns in Group A, it constitutes the second one for participants in Group B. Variability of the stimulus facilitates learning and induces significant increments in attentional arousal (Cooper and Aslin, 1989; Fernald and Kuhl, 1987; Trainor et al., 1997), which might be reflected in the greater reactivity to novel information observed in Group A. While more data should be gathered to better understand this phenomenon, the localization of the differential response in the right-lateralized areas further indicates that it pertains to the processing of vocal cues.

### Limitations

Some methodological and theoretical considerations merit attention. First, the length of the paradigm may have introduced a sampling bias, as more vulnerable infants who could not tolerate longer recording periods may have been excluded, and it also increases the likelihood of state changes. Newborn physiology changes rapidly, with behavioral transitions occurring within minutes, and such fluctuations can occur even in shorter experimental paradigms. Given recent evidence highlighting the distinct roles of sleep states in long-term cognitive development and functional connectivity (Lee et al., 2020; Uchitel et al., 2023), accounting for behavioral and physiological states during functional recordings should be a priority for future research (see Bastianello et al., 2025 for a comparative approach of sleep measures in infants).

A second consideration is that the present design did not include a control condition in which the interference word was spoken by the same speaker. However, a previous study employing a similar paradigm found that recognition does not persist under these circumstances (Benavides-Varela et al., 2011a). Since our protocol was more demanding, with shorter familiarization and longer retention, it is unlikely that a same-speaker interfering word would have yielded different results in our setting. Thus, due to practical challenges and ethical considerations associated with testing newborns, we did not include this condition. Incorporating such a condition in future studies could help further refine the interpretation of the interference effects observed in early memory formation.

### Conclusion

Understanding the mechanisms governing memory and the factors enhancing it is crucial for comprehending language development. This study assessed newborns' ability to retain a combination of speech sounds in the presence of acoustically novel interference. The findings showed that acoustic variability promotes separate memory traces of linguistic content rather than fully interfering with them. The presence of a new speaker may thus signal a new acoustic episode and facilitate the separation of linguistic memory traces. This suggests that the ability to encode information about the speaker is a fundamental process, potentially rooted in early brain mechanisms of cognitive development. This observation carries relevant implications when considered in relation to theories of memory and models of memory development (Alberini and Travaglia, 2017; Behm et al., 2025; Yates et al., 2025). Episodic memory is a multifaceted construct that, in its mature form, entails the ability to retrieve past events with contextual detail, typically involving autobiographical recollection and the integration of *what–where–when* information (Tulving, 1993). Our study does not aim to demonstrate the presence of a fully developed episodic memory system at birth, nor do we claim that newborns’ performance satisfies all hallmark criteria of mature episodic memory. We focused on sensitivity to speaker identity as a contextual dimension relevant to memory formation. Within this narrower sense, both the patterns of activation and the localization of the response provide evidence for early source–content binding (i.e. *what–who*), which can be considered a foundational aspect of episodic-like processing. Following up on this foundational step, future studies may track the gradual integration of additional aspects (*i.e. where-when*), ultimately leading to the maturation of a fully functional human episodic memory system, and investigate the neural mechanisms underlying this process.

## Methods

### Participants

Healthy full-term human newborns from a normal pregnancy (i.e. with no pathologies, perinatal, or neurological complications attested) were tested. Selection criteria included gestational age (GA) 37–42 weeks (range [37+1, 41+1]), Apgar scores ≥8 in the fifth and tenth minutes, absence of cephalohematoma or other conditions that could possibly affect cortical hemodynamics, intact hearing, head diameter within 32.5–37.0 cm range, and weight ≥2.5 Kg. Neonates were recruited from the Neonatal Care Unit of the Unit of Neonatology and the Obstetric Division of the University Hospital of Padova between May 2023 and September 2023. Informed consent for participation in the experiment was obtained from parents. The Ethics Committee for Clinical Research of the Province of Padova, Italy, approved the study. Thirty-two infants who provided good quality data were included in the study (18 females; age range [0, 4] days; mean weight 3.364 kg, SD 0.308 kg). Eleven additional neonates were tested but not included in the analyses due to fussiness (not even five blocks free of artifacts in at least one of the testing sequences; n=4), bad quality signal (more than 15 channels out of 42 marked as non-functional; n=6), and technical problems (n=1).

### Stimuli

Five pseudowords (CVCV structure, stressed on the first syllable) were used in the study (target and test words: /*mita*/, /*pelu*/, /*voli*/; interference words: /*noke*/, /*dafo*/). Two female speakers recorded the target/test words (/*mita*/, /*pelu*/, /*voli*/), while two male speakers recorded the interference words (/*noke*/, /*dafo*/). Pseudowords were edited using the open-source Praat software (Boersma and Heuven, 2001) to have a mean intensity of 70 dB and a duration of 700 ms. Detailed acoustic information can be found in the SI (Appendix 1—table 1).

### Procedure and data acquisition

Neonates were tested in a dimly lit hospital room while lying in their cribs (N=23) or mothers’ arms (N=9), in quiet rest or sleeping, to ensure their comfort and maintain an ecologically valid environment. Pseudowords were presented through two loudspeakers using the Psychopy software (Peirce et al., 2019), while fNIRS data were recorded using the NIRx NIRSPort system (light sources of 760 and 850 nm, maximum intensity 25 mW per fiber per wavelength). We designed a probe configuration with 16 sources and 15 detectors forming 42 channels. The optodes were positioned according to the 10–20 system, with locations selected using the devfOLD toolbox (Fu and Richards, 2021) to cover the IFG, STG, and PL (Figure 1A). The average distance between sources and detectors was 2.13 cm (range = [1.75, 2.62] cm, SD = 0.21 cm), and the sampling rate was 7.63 Hz.

The experiment consisted of a Familiarization phase, an Interference/Retention phase, and a Test phase. Each phase lasted 3 minutes and comprised five blocks. In each of the five blocks, six pseudowords were presented (inter-stimulus interval = 0.5–1.5 s; inter-block interval = 25–35 s; Figure 1B). The same pseudoword was presented in each phase.

A within-subject design was implemented by having two testing sequences separated by 9 min of silence: in Sequence 1, neonates heard the same word during familiarization and test (same-word condition; X u X), while in Sequence 2, a novel word was presented during the test phase (novel-word condition; Y w Z). The speakers and pseudowords were completely different in the two sequences. The pseudowords used in the different phases and the speakers were counterbalanced across participants. The order of the sequences was also counterbalanced across participants, resulting in Group A, presented with Sequence 1 and then Sequence 2, and Group B, presented with Sequence 2, followed by Sequence 1. Participants were initially assigned in a counterbalanced way to Group A or Group B, balancing the number of participants who completed the experiment across groups. Due to signal quality and attrition, the final sample consisted of 17 infants in Group A and 15 infants in Group B.

The paradigm was a modified version of a previously used experimental protocol (Benavides-Varela et al., 2011b; Benavides-Varela et al., 2011a; Benavides-Varela et al., 2012). The Familiarization phase was reduced from ten to five blocks based on previous data showing that five blocks already result in habituation (Benavides-Varela et al., 2017) and to accommodate the two sequences within a single testing session. In addition, the retention period was extended from two to three minutes.

### Data processing and analysis

#### Preprocessing

The first steps of data pre‐processing were performed using custom functions and functions of the Homer3 fNIRS package (https://openfnirs.org/software/homer/homer3/; Huppert et al., 2009) in Matlab 2024a. We first converted intensity to optical density using the Homer3 function hmrR_Intensity2OD and detected motion artifacts on optical density using a custom function. In brief, a copy of the data was created, and band-pass filtered between 0.01 and 0.7 Hz. Then, the maximum change in sliding time windows of 2 s (time step one sample) was computed, and a relative rejection threshold was obtained for each channel as $thresh\mathrm{=}q_{3}\mathrm{+}2\times \left (q_{3}\mathrm{-}q_{1}\right)$, where $q_{3}$ is the third quartile of the maximum changes distribution and $q_{1}$ the first quartile. Using relative thresholds results in a better trade-off between data recovery and artifact detection without needing to optimize the thresholds for each experiment and subject (Fló et al., 2022; Fló et al., 2019). Time windows with a maximum change above the threshold were rejected, obtaining a rejection/inclusion matrix (tIncCh_MotArt) of the same size as the data. The procedure was repeated thrice or until less than 0.5% of the data was rejected. Finally, a mask of 1 s was applied to the rejected data.

We used three metrics for channel pruning (i.e. defining non-functional channels): signal saturation, signal-to-noise ratio (SNR), and Scalp Coupling Index (SCI); for each of them, a matrix of the size of the data containing the metric per channel and sample was obtained. The saturation matrix was computed, marking saturated samples per channel when the intensity was outside the range [10^–6^, 2.5]. The SNR was computed in sliding time windows (length 5 s, step 2.5 s) as $SNR\mathrm{=}10\times log\left(\frac{mean(int)}{std(int)}\right)$, where *int* is the measured intensity. The matrix with the SNR was obtained based on the SNR in each time window. The SCI (Pollonini et al., 2014) was computed in sliding time windows (length 5 s, step 2.5 s) on the optical density band-pass filtered around the heartbeat frequency (heartbeat rate ±0.4 Hz). The SCI matrix was then obtained. The heartbeat was estimated using the fNIRS recording in sliding time windows (length 60 s, step 15 s) as follows: the optical density was band-pass filtered between 0.8 and 3.3 Hz, PCA was applied, and the autocorrelation was computed for the first principal component. Then, the cardiac frequency was estimated as $Card{\ }Freq\mathrm{=}{\ }\frac{1}{\delta }$, where $\delta $ is the time of the first peak of the autocorrelation –after the peak at zero-lag peak. The three metrics were evaluated on data segments free of motion artifacts for pruning channels (we call them *tInc_pruning*). t*Inc_pruning* segments were defined as those with less than 30% of the channels affected by motion artifacts and lasting at least 15 s (rejected segments shorter than 2 s were re-included). A channel was pruned if more than 30% of the samples included in *tInc_pruning* showed: (1) saturation, (2) SNR <15, or (3) SCI <0.6. Subjects with more than 15 out of 42 channels pruned were excluded from the analysis.

Artifact correction techniques can reduce artifacts’ size, but no meaningful data can be recovered if the duration of the artifact is longer than an HRF. Since infants' data might be contaminated with strong and long motion artifacts, we used the rejection matrix obtained from the artifacts detection step (*tIncCh_MotArt*) to define long segments heavily contaminated by motion and later reject blocks overlapping with them. These contaminated long-segments were defined as samples with more than 50% of channels contaminated with motion artifacts and lasting at least 10 s. Note that before the bad-segments definition, included segments lasting less than 5 s were also rejected. This decision was made because sandwiched periods (i.e. rejected-included-rejected usually correspond to fully bad segments where the rejection algorithm did not mark all as bad). We call the included segments *tInc*. Afterward, we corrected motion artifacts by applying Spline interpolation (Scholkmann et al., 2010) using the Homer3 function hmrR_MotionCorrectSpline (p=0.99), followed by Wavelet correction (Molavi and Dumont, 2012) using the Homer3 function hmrR_MotionCorrectWavelet (iqr = 1.5). Finally, we re-detected motion artifacts in the corrected data, and if new segments had more than 50% of channels rejected, they were marked as bad in *tInc*. A final rejection matrix (*tIncCh*) was obtained based on the last artifacts detection, saturation, SNR < 15, and SCI < 0.6, and later used to reject specific channels from included blocks.

Subsequent steps of the analysis were performed in Python using MNE (version 1.7.0) and MNE-NIRS (version 0.6.0; Luke et al., 2021). The data were band-pass filtered using an FIR filter between 0.01 and 0.3 Hz (transition bandwidths of 0.005 Hz for the high-pass and 0.1 Hz for low-pass) and converted to optical density using the modified Beer-Lambert law (partial path length factor 4.75; Scholkmann and Wolf, 2013). To obtain the HRF, data were segmented from –5 to 20 s relative to the onset of each stimulus block, linearly detrended, and baseline-corrected using the pre-stimulus interval. Channels for specific blocks were rejected if: (1) marked as bad during more than 50% of the block in the rejection matrix *tIncCh*, (2) had an outlier peak-to-peak signal change defined as $> q_{3}\mathrm{+}2\times \left (q_{3}\mathrm{-}q_{1}\right)$, computed on normalized data across channels and blocks. Blocks were rejected if: (1) overlapped with not included segments (i.e. tInc = 0), (2) had more than 35% of the active channels rejected. Subjects were rejected if more than 35% (more than 15 out of 42) of the channels were excluded from the recording (pruned channels). A testing sequence (familiarization/interference/test) for a given subject was excluded if fewer than five blocks were retained out of the 15 blocks (5 familiarization, 5 interference, 5 test). Of the 32 subjects with included data, 31 completed the same-word condition sequence, and 26 completed the novel-word condition sequence (25 both). On average, we obtained data for 22.3 subjects (range=[18, 27], std = 2.56) for each experimental block. The average number of blocks included for each phase (out of 5) across subjects was, for the sequence with the same word: 4.1 (SD 1.1) target blocks, 3.9 (SD 1.2) interference blocks, and 3.5 (SD 1.4) test blocks; and for the sequence with the novel word: 4.2 (SD 1.1) target blocks, 3.8 (SD 1.3) interference blocks, and 4.0 (SD 1.1) test blocks. The mean percentage of channels rejected for the whole recording among the included subjects was 5.21% (SD 6.66; range [0, 30.95] %).

The channel data were combined into six symmetric ROIs: IFG (left and right, each comprising 4 channels), STG (left and right, each comprising 5 channels), and PL (left and right, each comprising 7 channels; Figure 1A). On average across subjects and blocks, the activity for IFG left resulted from 4.0 channels (SD 0.16), IFG right 3.8 channels (SD 0.38), STG left 4.9 channels (SD 0.35), STG right 4.7 channels (SD 0.71), PL left 6.5 channels (SD 0.93), and PL right 5.4 channels (SD 1.58). The mean activity for each block over the time window [0, 15] s was used for statistical analysis. The time window was determined from the grand-average HRF across all blocks and subjects, which peaked at ~7 s from stimulus onset and returned to baseline at ~15 s (Appendix 1—figure 1).

### Statistical analysis

Changes in the concentration of oxygenated hemoglobin (HbO) and deoxygenated hemoglobin (HbR) were calculated. We used LMM for the analysis, with the mean activation as the dependent variable. Fixed effects were nested within the block number and the ROIs, while the participant was included as a random effect. The models were solved in R (version 4.2.1) using the lme4 package (version 1.1.31).

## Figures and Tables

**Appendix 1—figure 1.**
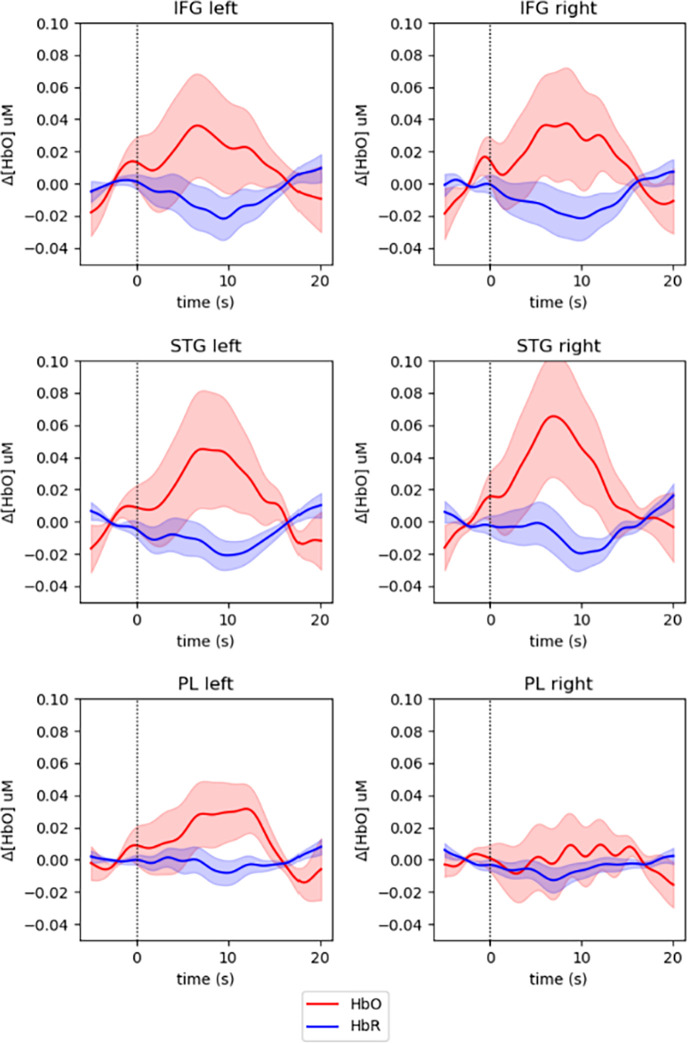
Grand HRF across all blocks. Shaded areas represent the standard error across subjects. HbO is represented in red and HbR in blue.

**Appendix 1—figure 2.**
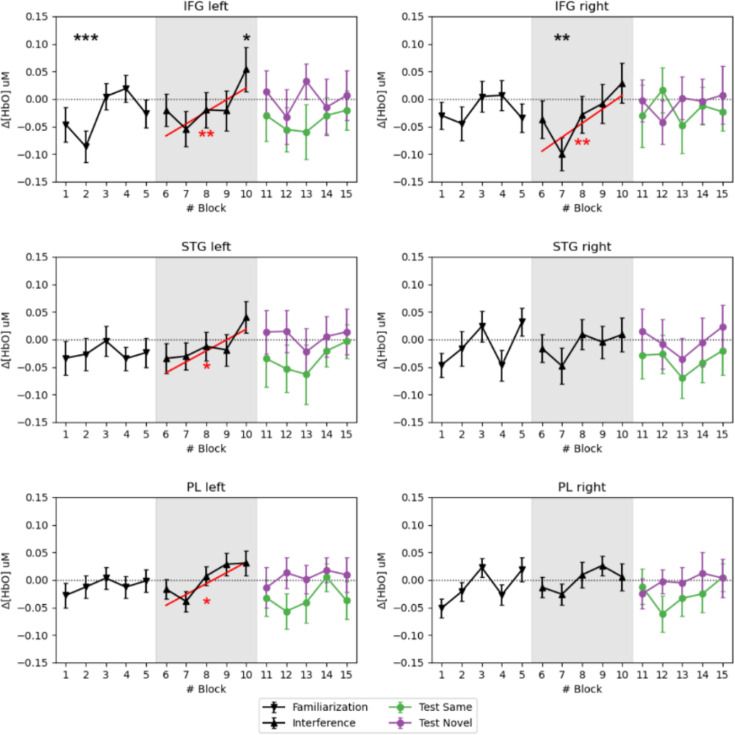
Block activity for HbR during the learning, interference, and test phases. Error bars represent the standard errors. The black continuous line depicts responses averaged across all participants and conditions. The same-word condition (green) and the novel-word condition (purple) are plotted in the test phase. The black asterisks during the learning and interference phases indicate that the response differed from zero. The red lines indicate a significant linear trend, as indicated by the red asterisks. No significant differences between conditions were observed (p>0.1).

**Appendix 1—figure 3.**
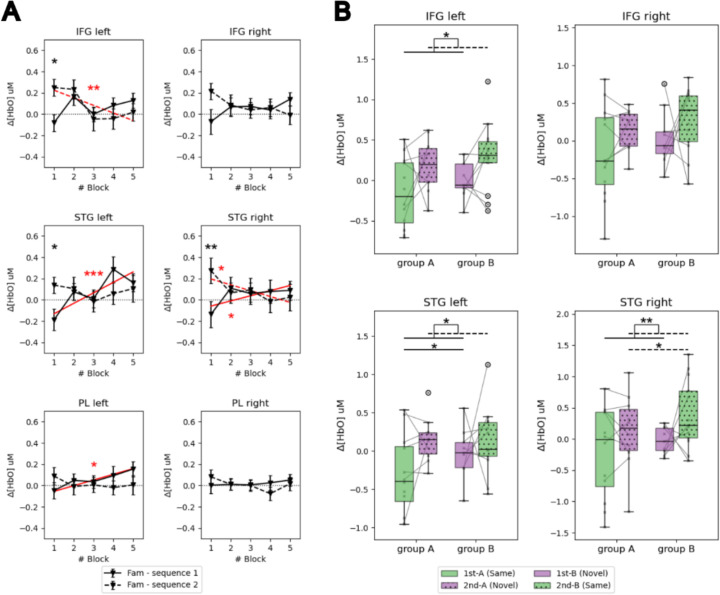
Differences in habituation responses during the familiarization phase across sequences. (**A**) Mean activity for HbO over blocks during the first (full-line) and second (dashed-line) familiarization phases. Error bars represent the standard errors. Asterisks indicate blocks showing significant differences between the first and second familiarization phases. The red lines indicate a significant linear trend, as indicated by the red asterisks. For the first familiarization, the LMM *act ~ –1+ROI + ROI:blocknumber + (1|sub)*, with *blocknumber* ranging from 0 to 4 showed a significant increase in activity over blocks in the left and right STG (left: slope = 0.098, SE = 0.024, p=0.00005; right: slope = 0.048, SE = 0.024, p=0.045) and left PL (slope = 0.053, SE = 0.024, p=0.027). During the second familiarization, the activity was higher than zero in the first block and decreased with block number on the right STG (intercept = 0.196, SE = 0.071, p=0.0066; slope = −0.055, SE = 0.026, p=0.033), and left and right IFG (left: intercept = 0.225, SE = 0.071, p=0.0019; slope = −0.071, SE = 0.026, p=0.0056). (**B**) Mean activity for HbO during the first block of the familiarization phase over STG and IFG, where major differences between the first and second testing sequence were observed. First sequence: full box; second sequence: dotted box. The colors indicate whether the familiarization belongs to the same or novel word testing sequence.

**Appendix 1—figure 4.**
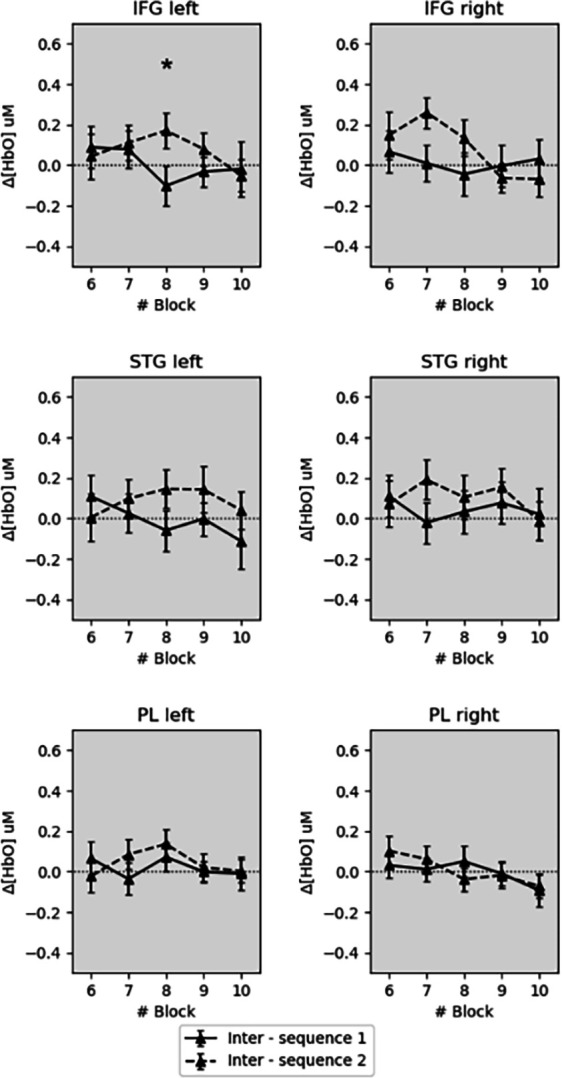
Habituation responses during the interference phase across sequences. Mean activity for HbO over blocks during the first (full-line) and second (dashed-line) interference phases. Error bars represent the standard errors. Asterisks indicate blocks showing significant differences between the first and second familiarization phases.

## Data Availability

The anonymized data collected are available as open data via the University of Padova online data repository: https://researchdata.cab.unipd.it/1403/ (DOI: https://doi.org/10.25430/researchdata.cab.unipd.it.00001403). The following dataset was generated: Benavides-VarelaS 2024MVM Study with newbornsResearch DATA UNIPD10.25430/researchdata.cab.unipd.it.00001403

## Additional files

MDAR checklist

## Appendix 1

### Methods

**Appendix 1—table 1.** Acoustic features across speakers. Analysis of acoustic features across speakers (familiarization and test pseudowords: voice_female_1; voice_female_2; interference words: voice_male_1; voice_male_2). Intensity and duration were held constant for each word (70 dB and 700 ms, respectively), while pitch (F0) and timbre (F1 and F2 of vowels) were extracted to assess acoustic differences across speakers.

mita	
*voice_female_1*	*voice_female_1*
pitch: 201.57 F1_mita_/i/: 376.65 F2_mita_/i/: 1249.95 F1_mita_/a/: 875.63 F2_mita_/a/: 1499.28	pitch: 188.43 F1_mita_/i/: 360.56 F2_mita_/i/: 2732.94 F1_mita_/a/: 865.07 F2_mita_/a/: 1369.88
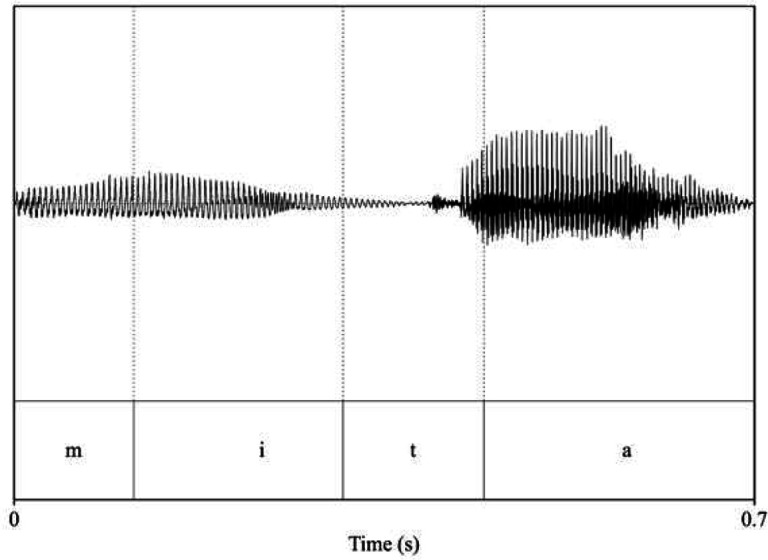
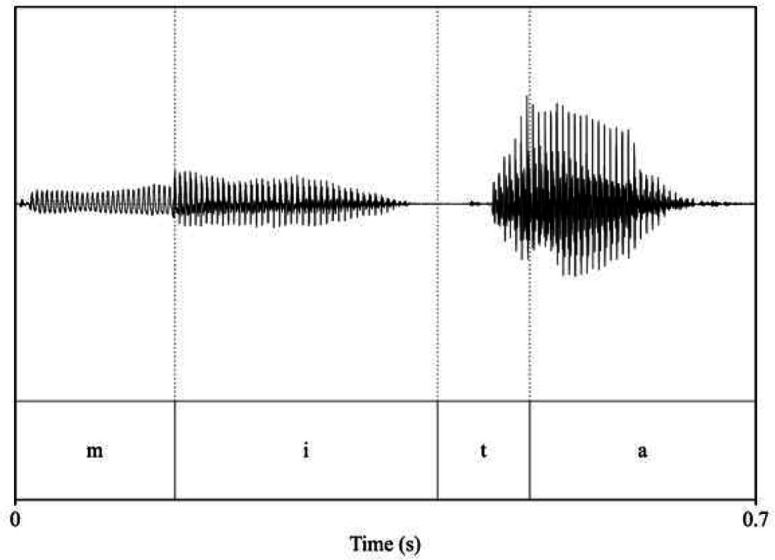
**pelu**	
*voice_female_1*	*voice_female_2*
pitch: 171.44 F1_pelu_/e/: 413.5 F2_pelu_/e/: 2441.28 F1_pelu_/u/: 405.49 F2_pelu_/u/: 822.86	pitch: 179.17 F1_pelu_/e/: 385.75 F2_pelu_/e/: 2480.84 F1_pelu_/u/: 376.49 F2_pelu_/u/: 837.6
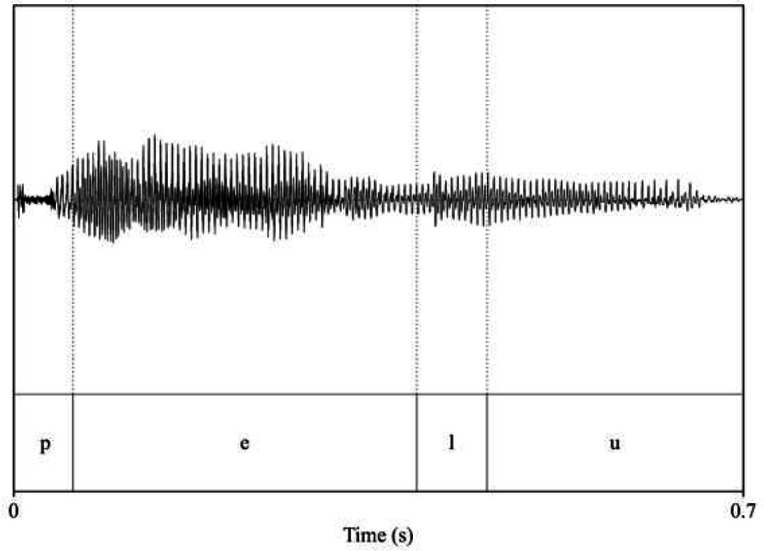
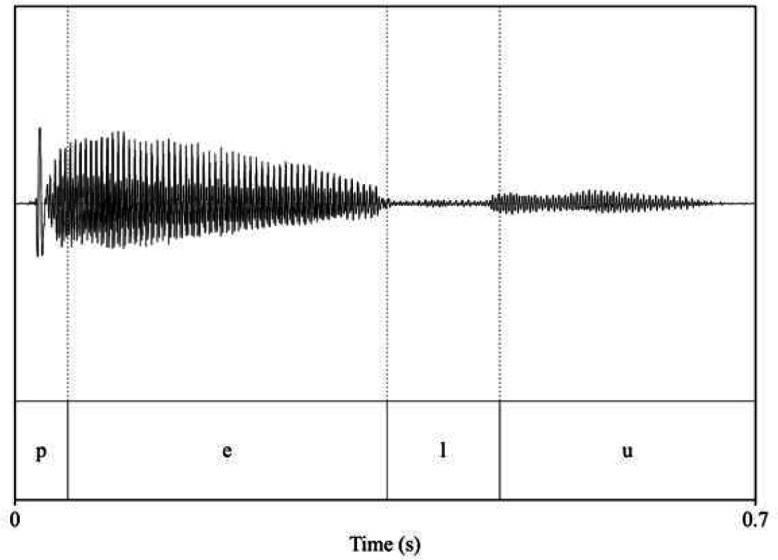
**voli**	
*voice_female_1*	*voice_female_2*
pitch: 193.75 F1_voli_/o/: 457.55 F2_voli_/o/: 908.18 F1_voli_/i/: 372.43 F2_voli_/i/: 2521.58	pitch: 173.96 F1_voli_/o/: 433.28 F2_voli_/o/: 870.12 F1_voli_/i/: 349.75 F2_voli_/i/: 2691.6
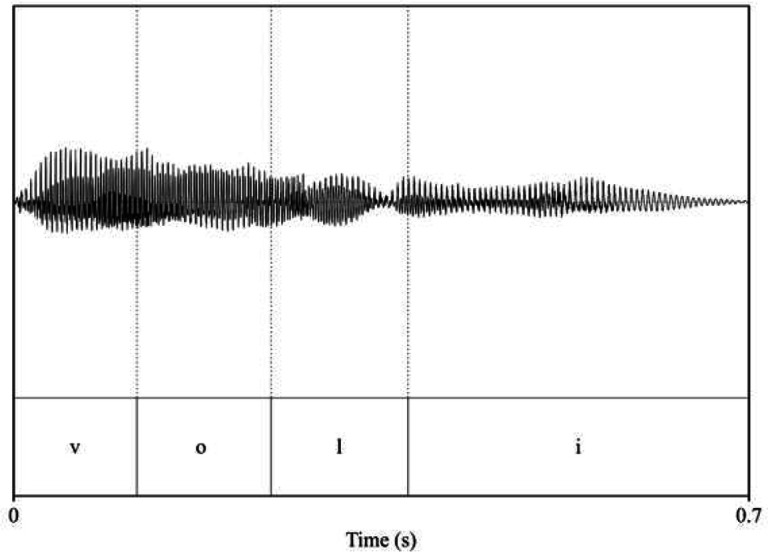
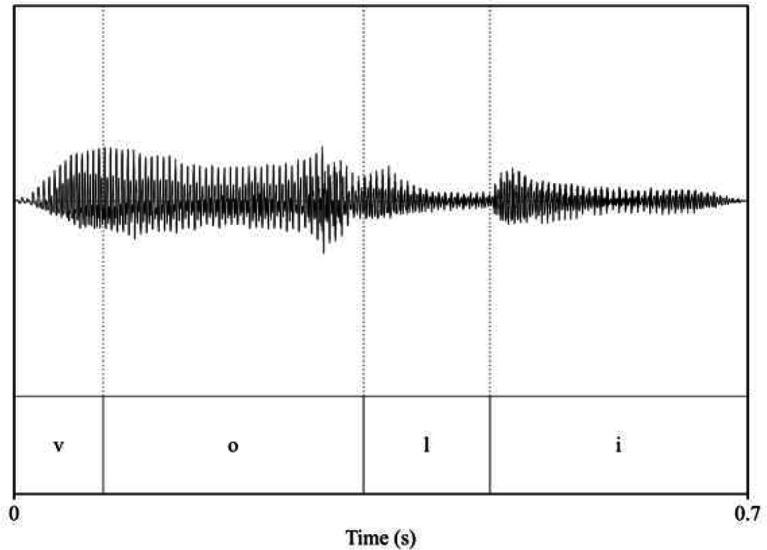
**dafo**	
*voice_male_1*	*voice_male_2*
pitch: 84.89 F1_dafo_/a/: 807.9 F2_dafo_/a/: 1126.4 F1_dafo_/o/: 551.35 F2_dafo_/o/: 1339.42	pitch: 86.12 F1_dafo_/a/: 676.91 F2_dafo_/a/: 1268.19 F1_dafo_/o/: 502.94 F2_dafo_/o/: 774.73
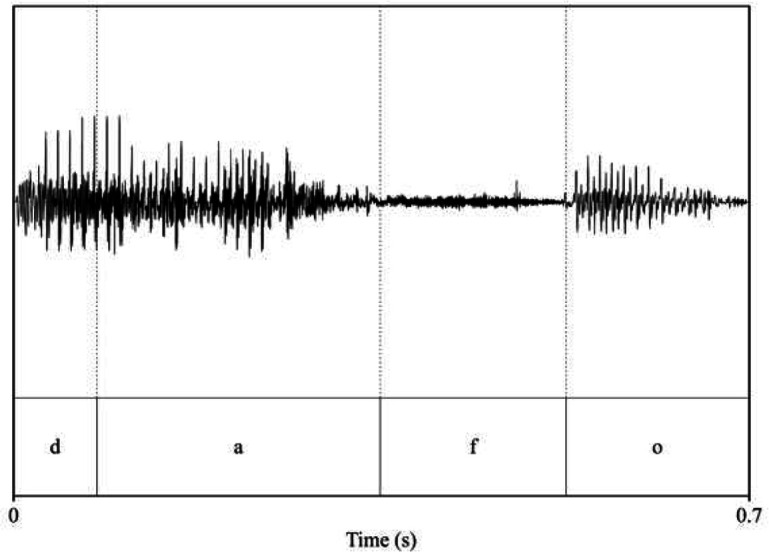
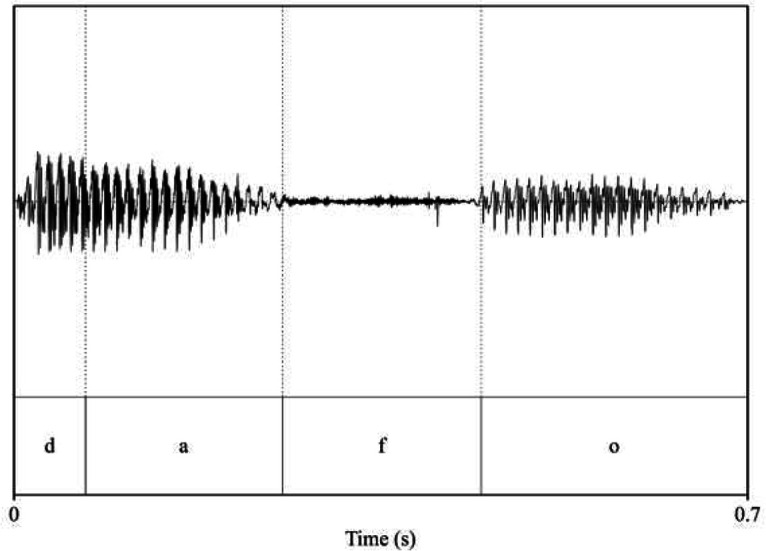
**noke**	
*voice_male_1*	*voice_male_2*
pitch: 85.92 F1_noke_/o/: 604 F2_noke_/o/: 1017.2 F1_noke_/e/: 488.63 F2_noke_/e/: 1901.05	pitch: 85.5 F1_noke_/o/: 593.86 F2_noke_/o/: 1018.64 F1_noke_/e/: 427.31 F2_noke_/e/: 1992.47
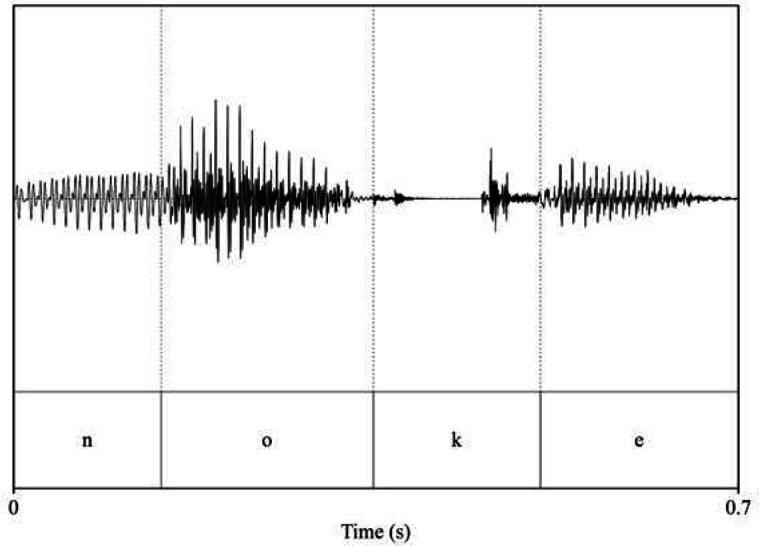
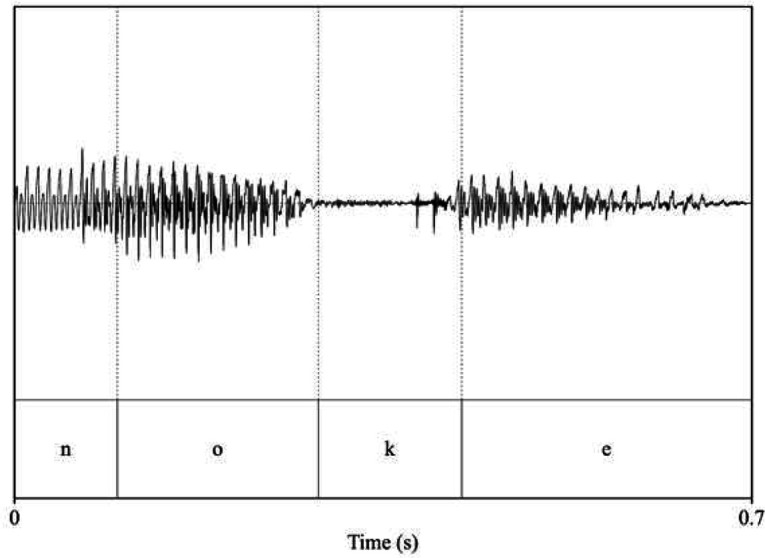

### Results

#### HRF response across blocks

We obtained the average response across all blocks in each ROI. This average response was used to define the time window for computing the average activity for each block. Since the grand average HRF returned to baseline level at ~15 s, we define the time window as [0, 15] s (Appendix 1—figure 1).

#### Results for HbR

##### Habituation and novelty effects

The LMM act ~–1+block:ROI + (1 | sub) during the learning phase, testing for activation differing from zero. There was deactivation in block 2 within left IFG (*β*=−0.0894, SE = 0.025, p=0.0004). During the interference phase, it showed deactivation in block 2 over right IFG (*β*=−0.0987, SE = 0.030, p=0.001) and activation in block 5 over left IFG (*β*=0.0686, SE = 0.029, p=0.017).

The LMM act ~–1+ROI + ROI:blocknumber+ (1 | sub), with blocknumber ranging from 0 to 4, showed no significant linear changes during the learning phase (p>0.05). Instead, during the interference phase, the activity increased over left IFG (intercept = −0.0663, SE = 0.030, p=0.027; slope = 0.0217, SE = 0.0082, p=0.0083), right IFG (intercept = −0.0946, SE = 0.030, p=0.0019; slope = 0.0253, SE = 0.0082, p=0.0021), left STG (intercept = −0.0602, SE = 0.030, p=0.047; slope = 0.0197, SE = 0.0082, p=0.017), and right PL (intercept = −0.0240, SE = 0.031, p=0.4; slope = 0.0192, SE = 0.0083, p=0.020).

Finally, no differences were observed between the activity of the last block of familiarization and the first block of interference (p>0.05).

##### Word recognition

The LMM act ~–1+block:ROI +block:ROI:condition+ (1 | sub) showed no significant differences between conditions for any block and ROI (p>0.1).

### Description of the LMM used for testing for order effects

Activation patterns might differ from the first to the second sequence because infants retain information from the first sequence that affects the processing of the second or due to changes in cognitive state. Groups A heard first the sequence [X y X] (*same-word* condition) and then [X y Z] (*novel-word* condition), while groups B heard [X y Z] followed by [X y X]. Thus, sequences and conditions are inversely linked in each group.

During the learning and interference phases, we were interested in investigating whether (1) there were main differences due to the position in time of the phases (first sequence presentation or second), (2) there were differences between the groups in sequence 1, and (3) there were differences between the groups in sequence 2. Differences between groups within each sequence might be due to individual differences. In the case of the second sequence, they might also be related to the different content that the two groups heard in the first sequence. To do so, we ran an LMM with sequence number and group as fixed effects and custom contrasts to produce interpretable results and test these hypotheses. The contrasts used were:

(H0.1) main effect of sequence:

$$\displaystyle \rm 0 = {(groupA_{sequence1}+groupA_{sequence1} - groupB_{sequence2} - groupB_{sequence2})}/{2},$$

The contrasts were nested within blocks and ROIs. The model was: act ~–1+block:ROI +block:ROI:C+ (1 | sub), where C corresponds to the contrasts. Therefore, a significant coefficient for contrast 1 signifies a main effect of sequence, while a significant coefficient for contrasts 2 and 3 implies differences between groups in sequences 1 and 2, respectively.

In the test phase, the group determines the conditions the participants hear in each sequence. Thus, we included the main effect of the condition in the LMM as contrasts of interest and added the differences between groups within each condition. Differences within conditions might, therefore, result from individual differences (group) or the test word appearing in the first or second sequence. The contrasts used were:

(H0.1) main effect of condition:

$$\displaystyle \rm 0 = (groupA_{novel} +groupB_{novel} - groupA_{same} - groupB_{same})/2,$$

(H0.2) differences between groups within the same-word condition:

$$\displaystyle \rm 0=groupA_{same} - groupB_{same},\, and$$

(H0.3) differences between groups within the novel-word condition:

$$\displaystyle \rm 0=groupA_{novel} - groupB_{novel}.$$

The contrasts were nested within blocks and ROIs. The model was: act ~ –1+block:ROI +block:ROI:C + (1 | sub), where C corresponds to the contrasts.

#### Activation patterns during learning

Appendix 1—figure 3 shows the activation patterns during the learning phase. Results are described in the main text.

#### Activation patterns during interference

The LMM *act ~ –1+block:ROI+(1|sub)* during the interference phase showed no significant differences (p>0.05), meaning activity did not significantly differ from zero in any block. The LMM testing for linear changes in activity *act ~ –1+ROI + ROI:blocknumber + (1|sub)*, with *blocknumber*, showed a positive intercept with a negative slope in the right IFG (intercept = 0.153, SE = 0.0684, p=0.026; slope = −0.0413, SE = 0.019, p=0.027), denoting initial high activity that decreased across blocks. Lastly, to investigate potential novelty effects arising from the transition from the learning to the interference phase, we compare the activity of the last block of the learning phase and the first block of the interference phase. No significant differences were observed in any ROI (p>0.1).

The analysis investigating order effects during the interference phase showed higher activity during the second sequence in the third block over the left IFG (*β*=−0.314, SE = 0.131, p=0.016). No significant differences were observed between groups in the first or second sequences (p>0.05).

#### Analysis at the channel level

To ensure that the selection of the ROIs did not have a substantial influence on the results, we ran the analysis testing for differences between conditions during the test phase at the channel level. To do so, we run the model act~–1+block:channel +block:channel:condition + (1|sub). The significant coefficients contrasting the conditions are shown in Appendix 1—table 2.

**Appendix 1—table 2.** Results for the analysis at the channel level, evaluating differences between conditions during the test phase. LMM: act ~ –1 + block:channel + block:channel:condition + (1|sub). Only significant coefficients (p<0.05) for the contrast between conditions are shown.

	Estimate	Std. Error	df	t value	Pr(>|t|)
block2:cha_S12_D12	0.409779	0.177042	7838.167	2.314586	0.020661
block5:cha_S12_D12	–0.55653	0.167365	7838.71	–3.32523	0.000888
block1:cha_S12_D13	–0.50074	0.165594	7837.931	–3.02393	0.002503
block5:cha_S12_D13	–0.36341	0.163359	7838.626	–2.22462	0.026135
block2:cha_S14_D12	0.500881	0.180004	7838.163	2.782615	0.005405
block5:cha_S14_D12	–0.43254	0.174754	7838.85	–2.47513	0.01334
block5:cha_S14_D13	–0.32359	0.164814	7838.501	–1.96335	0.049641
block2:cha_S14_D14	0.470902	0.184399	7838.162	2.553705	0.010677
block2:cha_S2_D1	0.431107	0.176339	7838.231	2.444757	0.014517
block2:cha_S2_D2	0.354384	0.170551	7837.98	2.077884	0.037753
block2:cha_S2_D3	0.382112	0.180017	7838.239	2.122645	0.033815
block1:cha_S2_D4	–0.34561	0.163692	7837.839	–2.11136	0.034773
block2:cha_S2_D4	0.50764	0.170551	7837.98	2.976476	0.002925
block2:cha_S4_D4	0.460346	0.170551	7837.98	2.699176	0.006966
block2:cha_S6_D4	0.536299	0.175077	7837.872	3.063223	0.002197
block2:cha_S6_D5	0.590803	0.172379	7838.074	3.427358	0.000613
block2:cha_S7_D5	0.420251	0.170551	7837.98	2.464085	0.013758
block2:cha_S8_D6	0.404044	0.170551	7837.98	2.369059	0.017858
block2:cha_S8_D7	0.360462	0.170551	7837.98	2.113519	0.034588
