# Supplementary material for: Verbal Episodic Processing in Newborns
Source: eLife. 2026 Jun 24;14:RP109096. doi: 10.7554/eLife.109096 (PMC13293605; doi:10.7554/eLife.109096)
Supplement: Peer review history

# Editor report: eLife Assessment

**DOI:** 10.7554/eLife.109096.3.sa0

**Authors:** Xing Tian

**Affiliations:** New York University Shanghai China

**Keywords:** Convincing, Fundamental

This **fundamental** study reports **convincing** evidence for early verbal episodic memory formation. The findings demonstrate that speaker identity is a crucial feature, enabling episodic-like memories from birth, and will be of interest to cognitive neuroscientists working on brain development, memory, language learning and social cognition.

# Referee report: Reviewer #1 (Public review)

**DOI:** 10.7554/eLife.109096.3.sa1

Summary:

This manuscript investigates whether newborns can use speaker identity to separate verbal memories, aiming to shed light on the earliest mechanisms of language learning and memory formation. The authors employ a well-designed experimental paradigm using functional near-infrared spectroscopy (fNIRS) to measure neural responses in newborns exposed to familiar and novel words, with careful counterbalancing and acoustic controls. Their main finding is that newborns show differential neural activation to novel versus familiar words, particularly when speaker identity changes, suggesting that even at birth, infants can use indexical cues to support memory.

Strengths:

Major strengths of the work include its innovative approach to a longstanding question in developmental science, the use of appropriate and state-of-the-art neuroimaging methods for this age group, and a thoughtful experimental design that attempts to control for order and acoustic confounds. The study addresses a significant gap in our understanding of how infants process and remember speech, and the data are presented transparently, with clear reporting of both significant and non-significant results.

A previous concern was that the recognition effect appeared restricted to a subgroup of participants. The authors clarify that the bilateral STG and left IFG effects were present in both groups - it was only the right IFG modulation that was group-dependent. This is an important distinction and is now clearer in the revised manuscript. The timing of the effect emerging in a specific testing window also appears less arbitrary given the authors' explanation that prior work guided the analytical approach, and that task difficulty was expected to determine whether recognition would appear in earlier or later test blocks.

The sample size question is handled honestly. A power analysis based on a related ANOVA study produced an implausibly small estimate of N=5-7, which the authors rightly set aside. Aligning with fNIRS neonate studies - where mean sample sizes around N=24 are standard - is defensible, and the within-subject design with mixed-model analysis does improve sensitivity relative to simpler approaches. This is now explained in the manuscript.

The episodic memory framing has been scaled back appropriately. The revised discussion is clear that the study demonstrates what-who binding - an early component of episodic-like processing - rather than mature episodic memory in the Tulvingian sense. This is a more honest characterization of what the paradigm can show, and it opens a reasonable developmental question about how the remaining components (where, when) come online over the first months and years of life.

Weaknesses

The weaknesses are largely interpretive rather than fatal to the core findings. The absence of a same-speaker interference control within the current paradigm means the causal role of speaker change cannot be established entirely from internal evidence alone - the inference relies partly on comparison with Benavides-Varela et al. (2011), which used a somewhat different design. This is a reasonable approach given the ethical and practical constraints of testing newborns, and the authors are transparent about it, but readers should keep in mind that the conclusion about speaker change as the critical variable is supported by converging evidence across studies rather than a direct within-study manipulation.

Overall, the study contributes new and meaningful data on an underexplored aspect of early speech processing: the role of the speaker as a contextual dimension in word memory. The findings, taken together with the prior literature, tell a coherent story and have real implications for theories of early language acquisition and the developmental origins of episodic-like memory. The paradigm is sound and the results are worth pursuing in larger and more controlled follow-up studies.

# Referee report: Reviewer #2 (Public review)

**DOI:** 10.7554/eLife.109096.3.sa2

Summary

Previous studies by some of the same authors of the actual manuscript showed that healthy human newborns memorize recently learned nonsense words. They exposed neonates to a familiarization period (several minutes) when multiple repetitions of a bisyllabic word were presented, uttered by the same speaker. Then they exposed neonates to an "interference period" when newborns listened to music or the same speaker uttering a different pseudoword. Finally, neonates were exposed to a test period when infants hear the familiarized word again. Interestingly, when the interference was music, the recognition of the word remained. The word recognition of the word was measured by using the NIRS technique, which estimates the regional brain oxygenation at the scalp level. Specifically, the brain response to the word in the test was reduced, unveiling a familiarity effect, while an increase in regional brain oxygenation corresponds to the detection of a "new word" due to a novelty effect. In previous studies, music does not erase the memory traces for a word (familiarity effect), while a different word uttered by the same speaker does.

The current study aims at exploring whether and how word memory is interfered with by other speech properties, specifically the changes in the speaker, while young children can distinguish speakers by processing the speech. The author's main hypothesis anticipates that new speaker recognition would produce less interference in the familiarized word because somehow neonates "separate" the processing of both words (familiarized uttered by one speaker, and interfering word, uttered by a different speaker), memorizing both words as different auditory events.

From my point of view, this hypothesis is interesting since the results would contribute to estimate the role of the speaker in word learning and speech processing early in life.

Major strengths:

(1) New data from neonates. Exploring neonates' cognitive abilities is a big challenge, and we need more data to enrich the knowledge of the early steps of language acquisition.

(2) The study contributes new data showing the role of speaker (recognition) on word learning (word memory), a quite unexplored factor. The idea that neonates include speakers in speech processing is not new, but its role in word memory has not been evaluated before. The possible interpretation is that neonates integrate the process of the linguistic and communicative aspects of speech at this early age.

(3) The study proposes a quite novel analytic approach. The new mixed models allow exploring the brain response considering an unbalanced design. More than the loss of data, which is frequent in infants' studies, the familiarization, interference and learning processes may take place at different moments of the experiment (e.g. related to changes in behavioural states along the experiment) or expressed in different regions (e.g. related to individual variations in optodes' locations and brain anatomy).

Main weaknesses:

I did not find major weaknesses. However, I would like to have more discussion or explanation in the following points.

(1) It would be fine to report the contribution of each infant to the analysis, i.e. how many good blocks, 1 to 5 in sequence 1 and 2, were provided by each infant.

(2) Why did the factor "blocknumber" range from 0 to 4? The authors should explain what block zero means and why not 1 to 5.

(3) I may suggest intending to integrate the changes in brain activity across the 3 phases. That is, whether changes in familiarization relate to changes in the test and interference phases. For instance, in Figure 2, the brain response distinguishes between same and novel words that occurred over IFG and STG in both hemispheres. However, in the right STG there was no initial increase in the brain response, and the response for the same was higher than the one for novels in the 5th block.

(4) Similarly, it is quite amazing that the brain did not increase the activity with respect to the familiarization during the interference phase, mainly over the left hemisphere, even if both the word and speaker changed. Although the discussion considers these findings, an integrated discussion of the detection of novel words and the detection of a novel speaker over time may benefit from a greater integration of the results.

Appraisal

The authors achieved their aims, because the design and analytic approaches showed significant differences. The conclusions are based on these results. Specifically, the hypothesis that neonates would memorize words after interference, when interfered speech is pronounced by a different speaker was supported by the data, in block 2 and 5 and discussed the potential mechanisms underlying these findings, such as separate processing for different speakers, likely related to the recognition of speaker identity.

I think the discussion is well structured, although I may suggest integrating the changes into the three phases of the study. Maybe comparing with other regions, not related to speech processing.

Evaluating neonates is a challenge. Because physiology is constantly changing. For instance, in 9 minutes newborns may transit from different behavioral states and experience different physiological needs.

This study offers the opportunity to inspire looking for commonalities and individual differences when investigating early memory capacities of newborns.

Comments on revisions:

The authors provided satisfactory answers to my concerns.

I recognize that, because of technical and ethical reasons, the studies with neonates are particularly challenging, however, with a well-balanced design as the one the authors applied, even with small samples the data constitute valuable sources to advance in the field.

Neonate brain works in a particularly state of intense metabolic, functional and structural changes, which we are far to understand. Current data contribute to fill this gap in knowledge.

# Author comment: Author response

**DOI:** 10.7554/eLife.109096.3.sa3

**Authors:** Emma Visibelli, Ana Fló, Eugenio Baraldi, Silvia Benavides-Varela

**Affiliations:** University of Padua Padova Italy; University of Padua Padua Italy; University of Padua Padova Italy; University of Padua Padova Italy

The following is the authors’ response to the original reviews.

**Reviewer #1 (Public review)**

Summary:

This manuscript investigates whether newborns can use speaker identity to separate verbal memories, aiming to shed light on the earliest mechanisms of language learning and memory formation. The authors employ a well-designed experimental paradigm using functional nearinfrared spectroscopy (fNIRS) to measure neural responses in newborns exposed to familiar and novel words, with careful counterbalancing and acoustic controls. Their main finding is that newborns show differential neural activation to novel versus familiar words, particularly when speaker identity changes, suggesting that even at birth, infants can use indexical cues to support memory.

Strengths:

Major strengths of the work include its innovative approach to a longstanding question in developmental science, the use of appropriate and state-of-the-art neuroimaging methods for this age group, and a thoughtful experimental design that attempts to control for order and acoustic confounds. The study addresses a significant gap in our understanding of how infants process and remember speech, and the data are presented transparently, with clear reporting of both significant and non-significant results.

Weaknesses:

However, there are notable weaknesses that limit the strength of the conclusions. The main recognition effect is restricted to a specific subgroup of participants and emerges only during a particular testing window, raising questions about the robustness and generalizability of the findings. The sample size, while typical for infant neuroimaging, is modest, and the statistical power is further reduced by missing data and group-dependent effects. Additionally, the claims regarding episodic memory and evolutionary implications are somewhat overstated, as the paradigm primarily demonstrates memory retention over a few minutes without evidence of the rich, contextually bound recall characteristic of fully developed episodic memory.

Overall, the authors have achieved their primary aim of demonstrating that speaker identity can facilitate memory separation in newborns, providing valuable preliminary evidence for early indexical processing in language learning. The results are intriguing and likely to stimulate further research, but the limitations in effect robustness and theoretical interpretation mean that the findings should be viewed as an important step forward rather than a definitive answer. The methods and data will be of interest to researchers studying infant cognition, memory, and language, and the study highlights both the promise and the challenges of probing complex cognitive processes in the earliest stages of life.

We thank the reviewer for their thoughtful and positive assessment of our work, and for giving us the opportunity to clarify points that may have been unclear in the original manuscript.

First, considering that the recognition response was quite consistent in previous studies, we expected the effect to emerge within a specific testing window, in either the first or the second block, depending on task difficulty. Accordingly, our analytical approach was designed to reflect this expectation, which was subsequently confirmed by the results. Second, the main recognition effect is not restricted to a specific subgroup of participants. Recognition responses were observed in both groups in the left IFG and bilateral STG. The only group-specific modulation was found in the right IFG, where the effect was primarily driven by Group A. This suggests that activity in this specific region may be influenced by contextual factors such as the nature and amount of recently processed stimuli. We have clarified these points in the revised manuscript to avoid the impression that the core effect is limited to a subset of participants or not generalizable across studies.

Regarding the sample size, a formal calculation was initially attempted based on the effect size reported in a closely related ANOVA-based study (Benavides-Varela et al., 2011; Study 2: Word recognition after intervening melodies, main effect for the comparison same vs novel word [F(1,26) = 19.318; *p*<0.0001 effect size f = .87)]. However, inputting this information into a dedicated software (G*power; α = 0.05; number of groups = 1; number of measurements = 2) leads to an estimated sample size of N = 5 to 7 (depending on the desired power, range = 0.800.95). This sample size is unrealistically small and not representative of current research standards in the field. A proper formal power analysis for the LMM is otherwise hard to perform, as we lack information about the expected variance and random-effects structure. We therefore aligned our sample size with prior newborn studies using similar stimuli and experimental designs, and with fNIRS studies in newborns and infants (for recent metanalysis see De Roever et al., 2018; Boek et al., 2023; Gemignani et al., 2023; which examined studies with mean N = 24; N range = 186 and sample sizes often including various conditions and groups). Note also that our design includes a within-subject comparison, our analytical approach models subject-level variance and handles unbalanced datasets and missing data (which are common in infant studies), thereby improving statistical sensitivity. We have now explicitly clarified this choice in the Introduction.

Finally, we revised the discussion to ensure that interpretations are aligned with our findings, by including a limitations section and a more explicit note regarding theories of memory.

Episodic memory is a multifaceted construct that matures over time through the integration of the what–who-where–when information. The present study does not aim to demonstrate the presence of a fully developed episodic memory system at birth; rather, it shows that specific features of episodic-like processing (i.e., what–who) are already bound from the first days of life. Future studies may track the progressive integration of additional episodic-related components leading to a mature episodic memory system.

**Reviewer #1 (Recommendations for the authors):**

(1) I wonder why a control condition with same-speaker interference was not included. Adding such a control would allow you to directly test whether the observed effects are truly due to speaker changes, rather than other acoustic or procedural factors. If it is not feasible to add this condition, please discuss its absence explicitly and clarify how it impacts the interpretation of your findings.

We thank the reviewer for raising the issue of a same-speaker interference control. A similar control has been tested previously using a closely related paradigm, showing that recognition does not persist when neonates hear another word produced by the same speaker during the retention period (Benavides-Varela et al., 2011). As noted in the manuscript, there were some methodological differences between that study and the current one. Most importantly, in the present study familiarization was reduced (from ten to five blocks) and the retention interval increased (two to three minutes), making the current paradigm more demanding. We reasoned that, if newborns forgot the word under the prior (less challenging) study, they would also forget it here if a same-speaker interference control would have been implemented. With the current manipulation, despite the difficulty of the paradigm, the recognition response was observed. This pattern suggests that speaker change, rather than general procedural factors, is central to the observed effect. Given these prior findings and the ethical constraints of testing newborns, we believe that adding a new same-speaker control is not essential. We have now made this rationale more explicit in the manuscript (discussion section, limitations, p. 16), hoping that this clarification will make our methodological choices clearer.

(2) It wasn't clear if Group A and Group B have the same number of infants, and whether they were randomly assigned. Please specify.

Participants were initially assigned to Group A or Group B in a counterbalanced way to maintain comparable group sizes. Due to attrition and subsequent exclusion for various reasons (e.g., low signal quality, fussiness, technical issues), the final sample consisted of 17 infants in Group A and 15 infants in Group B. We have now specified this information in the revised manuscript (p. 20).

(3) Please specify the exact number of fNIRS channels assigned to each region of interest (ROI), as it is currently difficult to map the channel numbers in Supplementary Table 2 to the optode montage shown in Figure 2. Additionally, report the percentage of usable channels after quality control.

The inferior frontal gyrus left and right ROIs comprised 4 channels each, the superior temporal gyrus left and right ROIs 5 channels each, and the parietal lobe left and right ROIs 7 channels each. This information has been added to the methods section, along with the average number of channels contributing to each ROI after data rejection and the percentage of channels rejected throughout the recording (p. 23).

(4) Also, a formal power analysis to justify your sample size would be helpful for evaluating the reliability of your findings and is increasingly expected in developmental neuroimaging research.

Thanks for this suggestion. As stated in the public response, we agree that power analyses constitute an important component of methodological rigor in the field. In our case, a formal calculation was initially attempted based on the effect size reported in a closely related ANOVAbased study (Benavides-Varela et al., 2011; Study. 2: Word recognition after intervening melodies, main effect for the comparison same vs novel word [F(1,26) = 19.318; *p*<0.0001 effect size f = .87)].

However, inputting this information into a dedicated software (G’power; α = 0.05; power range = 0.80-0.95; number of groups = 1; number of measurements = 2) leads to an estimated sample size of N = 5 to 7, which is unrealistically small and not representative of current research standards in the field. A proper formal power analysis for the LMM is otherwise hard to perform, as we lack information about the expected variance and random-effects structure. We therefore aligned our sample size with prior newborn studies using similar stimuli and experimental designs, and with fNIRS studies in newborns and infants for recent metanalysis see De Roever et al., 2018; Boek et al., 2023; Gemignani et al., 2023; which examined studies with mean N = 24; N range = 1-86 and sample sizes often including various conditions and groups. Note also that our design includes a within-subject comparison, and our analytical approach models subject-level variance and handles unbalanced datasets and missing data (which are common in infant studies), thereby improving statistical sensitivity.

(5) The manuscript references episodic memory explicitly in the abstract and introduction, emphasizing the role of speaker identity in enabling episodic-like memory from birth. However, this concept is not sufficiently addressed or delineated in the discussion. Episodic memory is generally understood as recalling events with contextual details, involving complex integrative processes that extend beyond simple recognition of auditory stimuli. Your paradigm demonstrates memory retention over a few minutes but does not provide strong evidence for the hallmark features of episodic memory, such as contextual binding or autobiographical recollection. Moreover, infant speech recognition and memory formation in early life are influenced by the immediacy and complexity of sensory input, which may not necessarily engage fully developed episodic systems. Clarifying these distinctions and making sure your interpretations and claims are consistent with them would enhance the conceptual clarity of the manuscript.

We agree that episodic memory is a multifaceted construct that, in its mature form, entails the ability to retrieve past events with contextual detail, typically involving autobiographical recollection and the integration of what–-who-where–when information (Tulving, 1993). Our study does not aim to demonstrate the presence of a fully developed episodic memory system at birth, nor do we claim that newborns’ performance satisfies all hallmark criteria of mature episodic memory.

Here, we focused on sensitivity to speaker identity as a contextual dimension relevant to memory formation. Within this narrower sense, both, the patterns of activation and the localization of the response provide evidence for early source–content binding (i.e., what–who), which can be considered a foundational aspect of episodic-like processing. Following up on this foundational step, future studies may track the gradual integration of additional aspects (where-when), ultimately leading to the maturation of a fully functional human episodic memory system.

We have now clarified this point in the revised manuscript (p. 17)

(6) Please add a dedicated limitations section. This should address the group-dependent nature of your main effects, the timing-specific recognition response, and any other methodological constraints that may impact the generalizability of your results.

We thank the reviewer for this comment. We have made our best to expose the limitations of our study in the text (p.16), specifically regarding the reasons for the lack of a control condition and the effects of frequent changes in sleeping states in newborns.

(7) Consider revising sections where claims may be overstated, particularly regarding episodic memory and evolutionary implications.

These sections have now been revised in the abstract and throughout the manuscript to ensure that interpretations remain proportionate to the data and consistent with current theoretical frameworks.

**Reviewer #2 (Public review):**

Summary:

Previous studies by some of the same authors of the actual manuscript showed that healthy human newborns memorize recently learned nonsense words. They exposed neonates to a familiarization period (several minutes) when multiple repetitions of a bisyllabic word were presented, uttered by the same speaker. Then they exposed neonates to an "interference period" when newborns listened to music or the same speaker uttering a different pseudoword. Finally, neonates were exposed to a test period when infants hear the familiarized word again. Interestingly, when the interference was music, the recognition of the word remained. The word recognition of the word was measured by using the NIRS technique, which estimates the regional brain oxygenation at the scalp level. Specifically, the brain response to the word in the test was reduced, unveiling a familiarity effect, while an increase in regional brain oxygenation corresponds to the detection of a "new word" due to a novelty effect. In previous studies, music does not erase the memory traces for a word (familiarity effect), while a different word uttered by the same speaker does.

The current study aims at exploring whether and how word memory is interfered with by other speech properties, specifically the changes in the speaker, while young children can distinguish speakers by processing the speech. The author's main hypothesis anticipates that new speaker recognition would produce less interference in the familiarized word because somehow neonates "separate" the processing of both words (familiarized uttered by one speaker, and interfering word, uttered by a different speaker), memorizing both words as different auditory events.

From my point of view, this hypothesis is interesting, since the results would contribute to estimating the role of the speaker in word learning and speech processing early in life.

Strengths:

(1) New data from neonates. Exploring neonates' cognitive abilities is a big challenge, and we need more data to enrich the knowledge of the early steps of language acquisition.

(2) The study contributes new data showing the role of speaker (recognition) on word learning (word memory), a quite unexplored factor. The idea that neonates include speakers in speech processing is not new, but its role in word memory has not been evaluated before. The possible interpretation is that neonates integrate the process of the linguistic and communicative aspects of speech at this early age.

(3) The study proposes a quite novel analytic approach. The new mixed models allow exploring the brain response considering an unbalanced design. More than the loss of data, which is frequent in infants' studies, the familiarization, interference and learning processes may take place at different moments of the experiment (e.g. related to changes in behavioural states along the experiment) or expressed in different regions (e.g. related to individual variations in optodes' locations and brain anatomy).

Weaknesses:

I did not find major weaknesses. However, I would like to have more discussion or explanation on the following points.

(1) It would be fine to report the contribution of each infant to the analysis, i.e. how many good blocks, 1 to 5 in sequence 1 and 2, were provided by each infant.

(2) Why did the factor "blocknumber" range from 0 to 4? The authors should explain what block zero means and why not 1 to 5.

(3) I may suggest intending to integrate the changes in brain activity across the 3 phases. That is, whether changes in familiarization relate to changes in the test and interference phases. For instance, in Figure 2, the brain response distinguishes between same and novel words that occurred over IFG and STG in both hemispheres. However, in the right STG there was no initial increase in the brain response, and the response for the same was higher than the one for novels in the 5th block.

(4) Similarly, it is quite amazing that the brain did not increase the activity with respect to the familiarization during the interference phase, mainly over the left hemisphere, even if both the word and speaker changed. Although the discussion considers these findings, an integrated discussion of the detection of novel words and the detection of a novel speaker over time may benefit from a greater integration of the results.

Appraisal:

The authors achieved their aims because the design and analytic approaches showed significant differences. The conclusions are based on these results. Specifically, the hypothesis that neonates would memorize words after interference, when interfered speech is pronounced by a different speaker, was supported by the data in blocks 2 and 5, and the potential mechanisms underlying these findings were discussed, such as separate processing for different speakers, likely related to the recognition of speaker identity.

I think the discussion is well-structured, although I may suggest integrating the changes into the three phases of the study. Maybe comparing with other regions, not related to speech processing.

Evaluating neonates is a challenge. Because physiology is constantly changing. For instance, in 9 minutes, newborns may transit from different behavioral states and experience different physiological needs.

We thank the reviewer for their constructive and positive appraisal of our work and for drawing attention to points that benefited from further clarification or discussion in the manuscript.

In the following, we address each point in turn, using the numbering of the reviewer’s identified concerns.

(1) In the Methods section (“Data Processing and Analysis”, p. 22), we have added detailed information about the number of data points contributed by each infant to the analyses.

(2) The factor “blocknumber” ranged from 0 to 4 for statistical purposes, allowing Block 0 to serve as the reference (intercept) in the model. This coding facilitated the interpretation of parameter estimates. We now clarify this in the revised manuscript (p. 7).

(3) Thanks for this relevant suggestion. In the Discussion, we now explicitly discuss the relationship across phases. We also acknowledged that a thorough examination of these issues lies beyond the scope of the present study as it will require future work based on multivariate and connectivity analyses.

(4) We thank the reviewer for this comment. In the revised manuscript, we have expanded the Discussion to clarify the absence of a strong novelty response during interference. The discussion highlights how the temporal properties of the hemodynamic response and the functional demands of each phase jointly shape the observable fNIRS signal in newborns, with purely sensory novelty effects likely increasing with maturation.

Finally, we agree that evaluating the transitions of sleeping states can further strengthen and clarify the results obtained in the present study. This has now been added as one of the limitations of this study.
